# Integrated healthcare services for HIV, diabetes mellitus and hypertension in selected health facilities in Kampala and Wakiso districts, Uganda: A qualitative methods study

**DOI:** 10.1371/journal.pgph.0000084

**Published:** 2022-02-03

**Authors:** Dominic Bukenya, Marie-Claire Van Hout, Elizabeth H. Shayo, Isaac Kitabye, Brian Musenze Junior, Joan Ritar Kasidi, Josephine Birungi, Shabbar Jaffar, Janet Seeley

**Affiliations:** 1 MRC/UVRI & LSHTM Uganda Research Unit, Entebbe, Uganda; 2 Public Health Institute, Liverpool John Moores University, Liverpool, United Kingdom; 3 National Institute for Medical Research, Dar-es-Salaam, Tanzania; 4 Department of International Public Health, Liverpool School of Tropical Medicine, Liverpool, United Kingdom; 5 Department of Global Health and Development, London School of Hygiene and Tropical Medicine, London, United Kingdom; University of the Witwatersrand, SOUTH AFRICA

## Abstract

Health policies in Africa are shifting towards integrated care services for chronic conditions, but in parts of Africa robust evidence on effectiveness is limited. We assessed the integration of vertical health services for HIV, diabetes and hypertension provided in a feasibility study within five health facilities in Uganda. From November 2018 to January 2020, we conducted a series of three in-depth interviews with 31, 29 and 24 service users attending the integrated clinics within Kampala and Wakiso districts. Ten healthcare workers were interviewed twice during the same period. Interviews were conducted in Luganda, translated into English, and analysed thematically using the concepts of availability, affordability and acceptability. All participants reported shortages of diabetes and hypertension drugs and diagnostic equipment prior to the establishment of the integrated clinics. These shortages were mostly addressed in the integrated clinics through a drugs buffer. Integration did not affect the already good provision of anti-retroviral therapy. The cost of transport reduced because of fewer clinic visits after integration. Healthcare workers reported that the main cause of non-adherence among users with diabetes and hypertension was poverty. Participants with diabetes and hypertension reported they could not afford private clinical investigations or purchase drugs prior to the establishment of the integrated clinics. The strengthening of drug supply for non-communicable conditions in the integrated clinics was welcomed. Most participants observed that the integrated clinic reduced feelings of stigma for those living with HIV. Sharing the clinic afforded privacy about an individual’s condition, and users were comfortable with the waiting room sitting arrangement. We found that integrating non-communicable disease and HIV care had benefits for all users. Integrated care could be an effective model of care if service users have access to a reliable supply of basic medicines for both HIV and non-communicable disease conditions.

## Background

Non-communicable diseases (NCDs), specifically hypertension and diabetes, now account for much of the global disease burden. The population of sub-Saharan Africa is experiencing a burden of infections and NCDs [1], albeit in the midst of social, political and economic uncertainties which influence health, disease and mortality patterns [2]. In East Africa, for example, a WHO STEPS [3] survey in south west Uganda and north west Tanzania reported diabetes and HIV prevalence to be 4% and 5% respectively, while hypertension prevalence ranged between 25% -30% [4]. Similarly, in Kenya the 2015 STEPS survey gave an age-standardised prevalence of hypertension of 24.5% with diabetes at 2.4% [5], and HIV prevalence in 2020 of 4.2% among adults aged 15–49 years [6].

The increasing availability of, and access to, HIV care and anti-retroviral treatment (ART) has enabled people living with HIV to live healthy lives into older age in most of the world [7–9]. People living with HIV and who are on ART can experience a range of NCD comorbidities, partly as a result of living longer when people become more prone to a range of non-communicable diseases [10]. However, in addition, HIV infection can increase the risk of some conditions, including cancer, and some ART drugs can increase the likelihood of heart disease and diabetes [11–14]. Therefore, this population is a part of a growing cohort in need of NCD care, requiring NCD services to be integrated into the care provided by HIV programmes [4, 15–18].

The World Health Organization (WHO), advocates for integration of health service delivery as the most logical way to provide health care [19]. The WHO defines integration as: `the organization and management of the health services so that people get the care they need, when they need it in ways that are user friendly, achieve the desired results and provide value for money’ [19]. In line with the WHO integration, we defined and operationalised integration as: ‘a one stop centre’ providing health care to the community and accessible to people with any chronic condition. Integration requires a well-trained work force, drug supplies, diagnostic resources and clinic structural improvements to support the successful treatment of a range of chronic conditions [13, 20, 21].

An integrated chronic care model was introduced in South Africa in 2011 to provide HIV and other chronic condition care in combined clinics [22]. The findings of feasibility studies showed that the model reduced service duplication and the frequency of patient visits in selected primary health facilities in South Africa [23–25]. Similar improvements were also found in HIV clinic settings in both Malawi [26] and Nigeria [27]. The integration of NCD care for people living with HIV has been highlighted as a need in Nigeria, Zambia, Swaziland and Kenya [28–30] as well as in Uganda [31–33].

Integration not only requires the implementation of supportive policies but also training for healthcare workers and infrastructure development, to enable changes in care delivery in public health systems [34–36]. Such provision is required for people with a range of chronic conditions to enable them to access integrated care in a single location. In Uganda, health provision for HIV and NCDs is usually offered separately through condition-specific clinics, and NCD care is less well funded than HIV care [37]. In addition, there is still a limited understanding of service user and provider perceptions and experiences of accessing, receiving, and providing integrated HIV and NCD care with which to inform the establishment and scaling up of integrated services. To respond to this need, we conducted a longitudinal study, using qualitative methods, to examine the perceptions and experiences of receiving and providing integrated HIV/NCD care in selected health facilities in Kampala and Wakiso districts in Uganda. Ultimately, we sought to understand better the integration process from diverse perspectives and experiences in order to contribute to the growing body of knowledge on integrated health services in Africa.

## Methodology

### Study background

This study was nested within a larger feasibility study entitled Management Of Chronic Conditions in Africa (MOCCA) which from August 2018 to December 2019 was conducted in five health facilities (four government and one private not for profit clinic) in Kampala and Wakiso districts in Uganda. The aim of MOCCA was to develop and evaluate a model of integrated HIV, hypertension and diabetes care where all health facility services were to be provided in a single location as a ‘one stop centre’. To facilitate this, the participating health facilities established integrated HIV/NCD clinics. Thus, patients with any one or more of the target conditions–HIV, diabetes or hypertension–were managed in a single clinic. This meant that people presenting with HIV, diabetes or hypertension all had the same waiting areas, were seen by the same clinical staff and used the same counselling, laboratory and pharmacy services.

Healthcare workers at the participating facilities were trained to ensure provision of quality and standardised care for all three conditions in an integrated manner. This was supported by the provision of a buffer supply of diabetes and hypertension drugs and laboratory supplies in the event of drug shortages in the Ugandan Government pharmaceutical supply chain. The MOCCA study did not supply buffer drugs for HIV treatment because the supply of these drugs through the Government system was dependable. The study also facilitated the identical file colouring for patients with all conditions in the HIV/NCD integrated clinic.

### Conceptual framework

**Fig 1:**
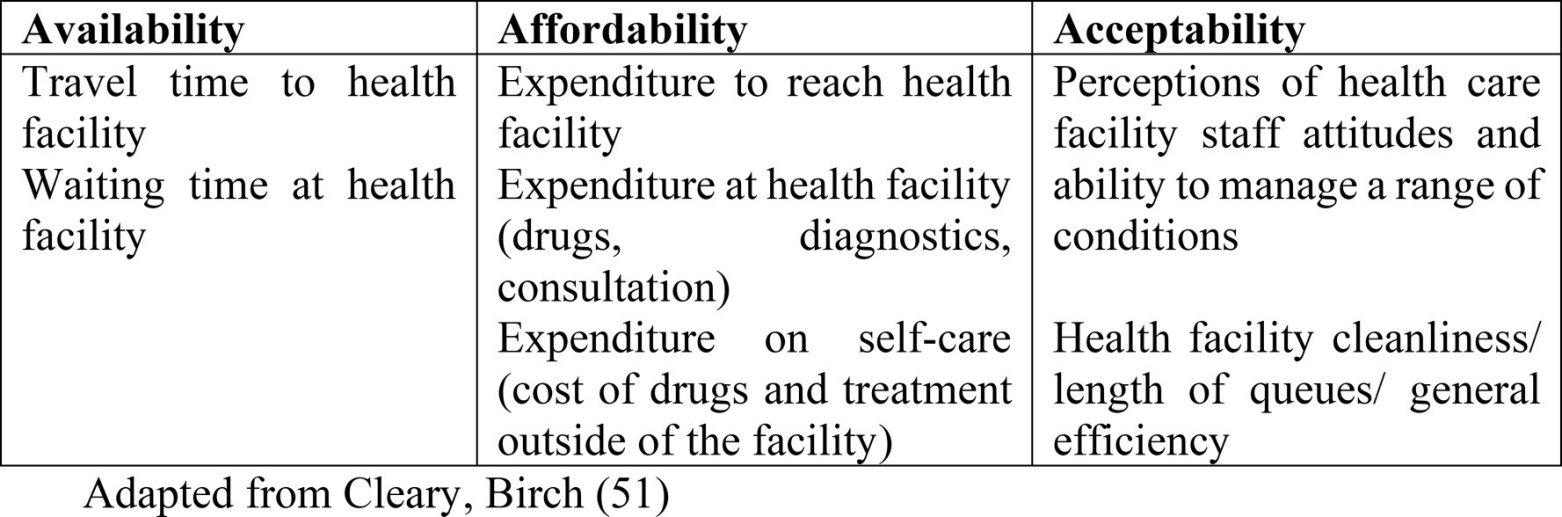
Dimensions of access.

A number of theoretical and conceptual frameworks have been applied in studies of integrated care systems which focus on concepts such as satisfaction and acceptability [38–41]. We utilised the conceptual framework developed and described by Thiede, Akweongo [42] and McIntyre, Thiede [43] on access to health care, since `access’ was a prominent concept arising from the data. Thiede, Akweongo [42] describe access as `the opportunity to use health services, reflecting an understanding that there is a set of circumstances that allows for the use of appropriate health services’ (p.104). They proceed to divide access into three dimensions: availability (physical access); affordability (financial access) and acceptability (cultural access). Cleary, Birch [44] further developed this framework to examine access to ART in South Africa, and defined the three dimensions in the following way: availability as the `fit between a patient’ needs and the type, place and time of services provided; affordability is the `fit between the costs of utilising a service and the patient’s ability to pay the associated costs’; and acceptability as the fit between healthcare provider and the patient’ attitudes and expectations of each other (p.142). We summarise these dimensions in Fig 1 below:

We used this framework to examine the dimensions of access to integrated HIV and NCD care in a Ugandan context.

### Study setting

The study took place in clinics at different levels in the Government system in Uganda. The five facilities included in the study were categorised as follows, and identified in this paper by letter:

A and B: Health centre IVs which have an inpatient capacity of 50–100 inpatient beds overseen by a medical doctor and offering the following services: general minor surgery, laboratory investigations, diagnosis and management of uncomplicated acute and stable chronic conditions including diabetes, hypertension and HIV. At these centres people with stable chronic conditions including diabetes, hypertension and HIV are treated (patients with complicated conditions are referred to district hospitals). Both facilities are in Wakiso District.

C and D: Health centre IIIs which are run by a clinician or a mid-wife and offer the following the services: diagnosis and management of uncomplicated chronic conditions including diabetes, hypertension and HIV, while referring complicated cases to health centre IVs. Both health facilities are in Kampala District.

E: Private not for profit facility which specialised in HIV care delivery and the treatment of uncomplicated diabetes and/or hypertension experienced by people living with HIV who attended the facility. This facility is in Kampala District.

All health facilities offered HIV and NCD care either in standalone clinics or in an outpatient department prior to MOCCA. The facilities were selected purposely to represent different health facility levels and ownership with a mix of urban and peri-urban characteristics.

The Medical Research Council /Uganda Virus Research Institute (MRC/UVRI) and the London School of Hygiene and Tropical Medicine (LSHTM), Uganda Research Unit, in collaboration with the Liverpool School of Tropical Medicine (LSTM) and the Uganda Ministry of Health, designed and implemented the feasibility study.

### Ethical considerations

The study received ethical clearance from The AIDS Support Organisation (TASO) Research Ethics Committee and Uganda National Council for Science and Technology (UNCST) in Uganda (Reference TASO REC/015/18-UG-REC-009) and the Liverpool School of Tropical Medicine in the United Kingdom (LSTM REC 18.044).

Permission to conduct the study was also sought from the relevant district and health facility authorities. At each clinic the social science interviewers provided information about the qualitative methods study embedded in MOCCA and sought informed consent. All participants (both users and HCW) signed an informed consent form before enrolment.

### Sample selection

Study participants were selected from health facility service users (hereafter referred to as `users’) enrolled or receiving care from the HIV, hypertension and diabetes clinics and healthcare workers (doctors, medical assistants and nurses, hereafter referred to as HCW) at the participating facilities. Users were purposively recruited at enrolment into HIV/NCD integrated care study clinic during the months of March and April 2019. We aimed to recruit five users with either HIV, hypertension, diabetes or a combination of these conditions from each participating facility. Out of the five, we recruited two who had a combination of either HIV with hypertension or diabetes or had hypertension and diabetes. The three other users recruited were to have been diagnosed with one of the three conditions (HIV, diabetes or hypertension). The focus for recruitment was the condition and a combination of conditions rather than age or sex. We recruited/sampled additional individuals because we anticipated loss to follow up. By the end of the recruitment period, a total of 31 users had been recruited into the study. Two HCWs (nurses, other clinicians and in-charges of the clinics) were purposively selected from each site at the beginning of the study to take part in provider interviews. The study aimed to recruit HCWs engaged in HIV and NCD care delivery. In total ten HCWs were recruited. The person in charge of each facility supported the selection of user participants and the two HCWs from each facility.

### Data collection

Data collection was undertaken from November 2018 to February 2020. Service users were recruited sequentially and interviewed after their visit to the clinic. Users were interviewed prior to the establishment of the HIV/NCD integrated clinic (Phase 1: March–April 2019), two to eight weeks after enrolment (Phase 2: June-July 2019) and nine months after recruitment, at the end of the integrated clinic feasibility study (Phase 3: January–February 2020).

HCWs had two rounds of interviews. The first was carried out prior to the establishment of the integrated clinic between May–June 2019. The second round was November 2019-January 2020 at the end of the integrated clinic follow up time.

Interviews were used to investigate users’ perspectives prior to the establishment of the NCD integrated clinic, soon after its establishment and at the end of the feasibility study. The same technique explored HCWs’ perspective prior to establishment of the integrated clinic and at the end of feasibility study. User and HCW interviews were supported by clinic level observations.

Observations of day-to-day practice in each facility, to observe waiting time, room and service access procedures were undertaken three times: in November 2018 prior to the establishment of the integrated clinic at the participating sites, then in June 2019 and finally November 2019 (see S1 File).

At the first round of interviews, users were asked about the services they expected to be provided with at the HIV/NCD integrated clinic (S1 File–guides in English and Luganda). Subsequent interviews explored the experiences attending the HIV/NCD integrated clinic. In brief, the phase two and three interview guide covered: patient demographics, health conditions for which care was sought, health seeking since the HIV/NCD integrated care establishment, procedure followed while seeking integrated HIV/NCD care, quality of services received, comfort with the sitting arrangement at the HIV/NCD integrated clinic and appropriateness of the HIV/NCD integrated clinic care. Additional questions included: cost of accessing integrated HIV/NCD care, freedom of entry, discussion and movement around the health facility among others (see S1 File).

The HCW interview guide explored conditions treated at their health facilities, types of HIV/NCD services offered, what HCWs did individually and at an institutional level to ensure provision of user-friendly services. Other topics included: changes in the management of HIV, diabetes and hypertension in the previous six months, what had to be done to ensure user-friendly service provision at individual/institution levels. HCWs were also asked about patient attitudes and perceptions on the NCD integrated clinic (S1 File–guides in English only). The final HCW interview, explored changes introduced in the HIV and NCD care delivery in the previous six months, HCW thoughts and opinions on what would be the benefits and challenges of integrated care delivery; HCW satisfaction with the integrated care delivery; user views about integrated services, and what needed to be done to ensure increased demand for integrated HIV and NCD care (S1 File).

The clinic observations which documented changes at the clinic levels and were used to inform the implementing team of any adaptations required. Observations investigated how services were provided in practice in standalone clinics (pre-phase 1) and were used to identify strengths, weaknesses and lessons learnt from the integrated HIV/NCD clinic. Baseline observations investigated experiences of NCD care delivery in the standalone condition-specific clinics, while subsequent data collection covered the experiences of the integrated clinics.

### Data management

All interviews were conducted by trained and experienced social science interviewers in a private place at each clinic. All interviews were face to face and audio-recorded after obtaining consent from the study participant. Interviews with users were conducted in Luganda, the main local language in the study area while those with HCWs were conducted in English (see S1 File). Interview transcription was done verbatim and later translated into English for the user interview transcripts. Each interviewer proof-read their transcripts to ensure completeness and accuracy. The first author reviewed the transcripts throughout the course of the study to check interview content and format. Where gaps were identified in the transcripts, the audio recordings were listened to, to check the reason for the gap and to confirm the correctness of the transcription. Final transcripts were kept on a password protected study computer, and organised in a study folder with sub folders for each data category. The data were backed up on a secure server.

### Data analysis

The first author read all the transcripts several times to fully understand the material. Through this process, data patterns were observed and emerging themes were identified, shared, discussed and agreed with the MCH, EHS, SJ and JS. The first author grouped the emerging themes and patterns according to the constructs of the access conceptual framework described above to develop the coding framework. The coding framework was discussed and agreed upon by DB, MCH, EHS, IK, BMJ, JRK and JS. The first author manually coded all the data in a way that allowed comparison for differences and similarities at different data collection points using deductive and inductive processes. Illustrative quotes in the participants own words were extracted from the interviews and are presented in this paper. Data from users, healthcare providers and the observations were triangulated to increase the richness and trustworthiness of the findings. The entire analysis process involved back and forth discussions between DB, MCH and JS.

**Fig 2:**
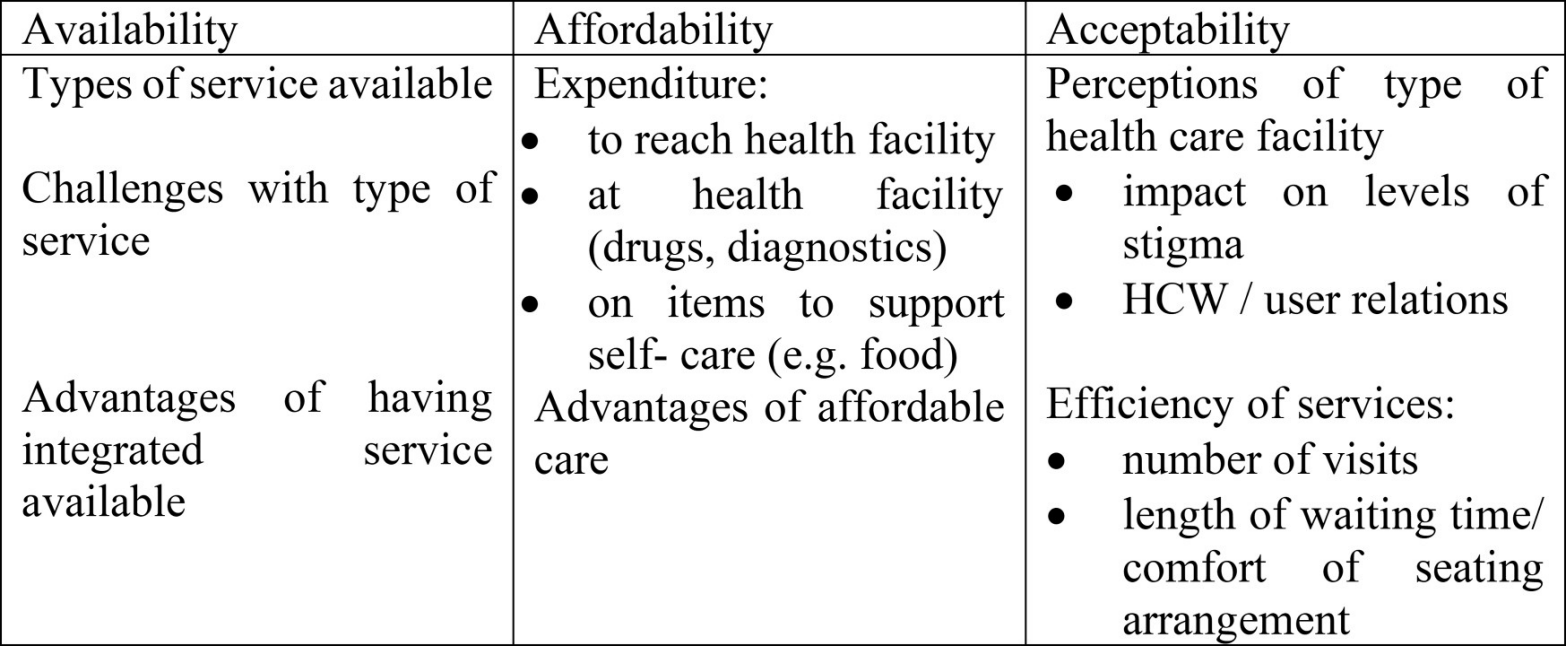
Dimensions of access to integrated and non-integrated care services.

Our findings presented in this paper are grouped by the three dimensions of access: availability, affordability and acceptability. We have grouped the themes derived from the data during analysis under each of these headings, following the example of Cleary, Birch [44], having adapted their framework for our study (see Fig 2 below).

## Results

Demographically. the 31 users had a mean age of 45 years (range of 23–72 years) and most (22 out of 31) were women. Slightly more than half were married, educated up to secondary level and earned a living through informal businesses. In terms of their health, most users had hypertension and diabetes (Table 1). HCWs had a mean age of 36 (range 28–51) years, most were women (six), married and educated up to tertiary level (Table 2).

**Table 1:** User study profile participants.

Variable	Categories	Phase I	Phase II	Phase III
Sex				
	Male (%)	9 (29)	7 (24)	5 (21)
	Female	22 (71)	22 (76)	19 (79)
Age (years)				
	Mean (SD)	45.1 (13.81)	44.9 (12.3)	44.1 (11.8)
	Range	23–72	23–72	24–72
Education level				
	No education	3 (10)	2 (7)	2 (8)
	Primary	10 (32)	10 (34)	7 (30)
	Secondary	16 (52)	15 (52)	13 (54)
	Tertiary	2 (6)	2 (7)	2 (8)
Marital status				
	Married	17 (55)	17 (58)	15 (62)
	Separated/divorced	5 (16)	4 (14)	3 (13)
	Single/widow	9 (29)	8 (28)	6 (25)
Livelihood/income sources				
	Business/informal	18 (58)	16 (55)	13 (54)
	Formal	3 (10)	3 (10)	3 (13)
	Unemployed	10 (32)	10 (35)	8 (33)
Disease condition				
	Hypertension	10 (32)	8 (28)	5 (21)
	Diabetes	8 (27)	8 (28)	6 (25)
	HIV	7 (22)	7 (24)	7 (29)
	Multi morbidity	6 (19)	6 (20)	6 (25)

**Table 2:** Healthcare worker profile study participants.

Variable	Categories	Phase I	Phase II
Sex			
	Male (%)	4 (40)	3 (33)
	Female	6 (60)	6 (67)
Age (years)			
	Mean (SD)	36 (37)	36 (38)
	Range	28–51	28–51
Education level			
	Tertiary	6 (60)	6 (67)
	University	4 (40)	3 (33)
Marital status			
	Married	7 (70)	6 (67)
	Single	3 (30)	3 (33)

We now present the findings on the three dimensions from our conceptual framework, availability, affordability and acceptability in turn, and divided between `before integration’ and `after integration’ for each dimension.

### Availability

#### Type of services available before the establishment of the integrated HIV/NCD clinics

All users reported that the health facilities provided HIV, diabetes and hypertension care prior to the establishment of the integrated HIV/NCD clinic. HIV care was similar in all the participating health facilities and included: pre and post-test counselling, anti-retroviral therapy initiation and drug refills, coupled with adherence counselling. Neither HCWs nor users reported experiencing HIV drug shortages or problems accessing diagnostic testing materials.

*Each time*, *I have come to the facility for HIV drugs refill*, *I get all the drugs assigned [prescribed]*. *There has never been a day I found when there were no drugs*. (user #7, 42-year-old male with NCD and HIV, facility D, Phase 1).

Although the participating public facilities provided diabetic and hypertensive care together in an NCD clinic, the NCD care provision was often subject to drug and/or reagent shortages coupled with missing or malfunctioning diagnostic equipment.

*It is the diabetes and hypertension drugs that are often unavailable*. (user #29, 63-year-old male with NCD and HIV, facility A, Phase 1).

However, this was not the case in the private facility (E), where no user reported experiencing drug shortages, or laboratory reagents missing or malfunctioning diagnostic equipment for HIV or NCD conditions.

The users attending all health facilities prior to integration were familiar with the services available to them for their treatment needs.

*They give us health education sessions every time we visit the facility*. *These health education sessions are given by both men and women and usually we are seated in a mixed up [more than one NCD] arrangement*. *Then I go to see the ART doctor who reviews my files and after that is when I come to get drugs*. (user #4, 60-year-old female with NCD and HIV, facility B, Phase 1).

Treatment services for the HIV and NCDs were in different places in the facility.

#### HIV/NCD service availability after the establishment of the HIV/NCD integrated clini

All HCWs reported that the establishment of the integrated clinic boosted the availability of diabetes and hypertension care, noting that HIV care availability was already sufficient. They further explained that shortly before the establishment of the integrated clinics, their facilities were supplied with new functional diagnostic equipment for diabetes and hypertension, such as blood pressure machines and dip sticks. They added that a diabetes and hypertension drug buffer stock was also established to cover for drug shortages. These augmented services were, as we explain above, provided as part of MOCCA. It is not surprising therefore, that it was during the second round of interviews the HCWs reported improved diabetic and hypertensive drug availability. In Facility D two clinical staff, both women aged about 30 years, explained how the care provision had been transformed:

*We have got medical equipment like blood pressure (BP) machines from the project*. *The project provided four BP machines*, *batteries and glucometers*. *Without these*, *we wouldn’t be able to test the patients without testing strips*. *We used to treat diabetes and hypertension before the integration but not as intensively as we do now*. *The project also provided some drugs for hypertension and diabetes and this has increased drug availability for patients*. (HCW #8, Phase 2).

*Previously there was a challenge with drug availability as drugs would always be out of stock*, *but after integration*, *drug availability improved greatly and this built confidence among the patients and even the retention levels became higher*. (HCW #2, Phase 2).

Almost all diabetes and hypertension users at the public health facilities also reported that drug shortages of drugs for those conditions had become rare since the establishment of the integrated clinic.

*It [integration] was good because I used to come to the facility and they could still prescribe the drugs for me and ask me to go and buy them from elsewhere*. *I now get free care and treatment as well*, *unlike back then*. (user #23, 50-year-old female with NCD, facility C, Phase 2).

However, as MOCCA was coming to an end (when the third round of interviews took place) there were a few users with diabetes and hypertension who reported that drug shortages had slowly started creeping back.

*I don’t pay any cost for healthcare services at this facility except [now] I only get one type of the prescribed drugs and I buy the other*. (user #14, 36-year-old female with NCD, facility D, Phase 3).

The participants who were accessing integrated care from facility E, the private not for profit facility, did not report experiencing any NCD drug shortages at any time during the follow up period. However, users of this clinic did report that the establishment of the integrated clinic marked the start of active screening of diabetes and hypertension among the people living with HIV attending that facility. Those users described how active screening for diabetes and hypertension for them accompanied the opening up of the facility to users accessing non- HIV-care.

*Because of the integration*, *I can now be screened and treated for diabetes and hypertension at my HIV clinic but previously I have been going to private clinics to test for those diseases*. (user #11, 31-year-old female with HIV, facility E, Phase 3).

#### Challenges with types of service before establishment of integrated HIV/NCD clinic

As the users of facility E note above, prior to establishment of HIV/NCD integrated clinic, that facility provided limited diabetes and hypertension care to users in receipt of HIV care. Complicated diabetes and hypertension cases at that facility were referred to the neighbouring national referral hospital.

In the public health facilities, the HCWs reported during the first round of interviews that the availability of diabetic and hypertensive care was limited due to drug and diagnostic material shortages, and faulty or missing diagnostic equipment.

*There are some challenges when the facility runs out of stock for some diagnostic instruments such as testing kits and sometimes it could take almost three months without testing clients*. *There are times when a blood pressure machine also breaks down and this could go on for even a week*. *This makes the clinic miss out on some very important measurements that would have been captured [*…*] when you ask the client to go and do the test out there*, *some clients are not in position to*. (HCW #6, 29-year-old male, facility C, Phase 1).

Users in the public health facilities reported that often, when they had sought care for diabetes and hypertension, they were given prescriptions and advised to go and buy the drugs from private pharmacies.

However, in one of the facilities (A), the HCWs had initiated a diabetes and hypertension club where users contributed some money towards their refill visits. The diabetes and hypertension users at this facility explained this money was used to buy themselves buffer drugs/testing materials to counter the shortages.

*I pay Shs 30*,*000shs [US Dollars 8] every six months in the [user] club and this money is used for buying testing strips and drugs in case of stock outs*. *This method just started recently in the previous years we used to go without drugs when the government drugs were out of stock*. (user #16, 48-year-old female with NCD, facility A, Phase 1).

The HCW role was only to provide guidance on where to buy the drugs for the buffer stock financed and managed by the users themselves.

### Affordability

#### Expenditure to reach health facility before the establishment of the HIV/NCD integrated clinic

Regardless of the disease condition, most users reported that prior to the establishment of the HIV/NCD integrated clinic their inability to afford transport to and from the health facilities hindered their access to care. The challenge of finding money to support a visit to the clinic was a recurrent theme throughout the study. This was unsurprising since most users continued to attend the facility they used prior to integration.

*The distance is long and I use 2*,*000 shillings [~ one USD] to go to the facility but I find it hard to get money for transport*. (user #11, 31-year-old female with HIV, facility E, Phase 1).

*I sometimes fail to get transport to the facility for treatment*. *I came with a boda boda [motorcycle taxi] which costs me Shs 2000 [~ one USD] to come to the health facility*. *I am thinking of walking back home in the guise of doing exercise because I have no money at hand*. (user #26, 33-year-old male with NCD and HIV, facility A, Phase 2).

Some HCWs agreed that transport was a major challenge to accessing health care for all users in general and was not only limited to those coming for HIV, diabetes and hypertension care. Lack of transport was a common reason for people to miss drug refills and clinic appointments.

*I have a concern some clients miss their appointment and never show up*, *this is common for HIV clients*. *There are some clients of hypertension and diabetes who do not take these drugs [as being] very important and serious*. *[…] some say they do not have transport since some clients come from very far*. (HCW #7, 51-year-old female, facility C, Phase 1).

A small number of users reported no challenges with transport because they lived close to the facility where they were accessing care.

*The facility is not so far from where I stay*. *The distance is walkable*. *I use a boda boda [motorcycle taxi] if I am late and that would cost me about Shs 2*,*000 [~ one USD] but usually I move on foot and that takes me about an hour*. (user #17, 46-year-old male with HIV, facility C, Phase 1).

Integration reduced the number of visits users had to make to facilities (as we discuss further below), which did make accessing care more affordable overall, even if finding funds for each clinic visit remained challenging.

#### Expenditure on drugs, diagnostics and consultations before establishment of the HIV/NCD integrated clinic

Prior to the establishment of the integrated clinic, diabetes and hypertension users reported an inability to buy diabetic and hypertensive drugs from private pharmacies. In addition to this, some diabetes users reported that they could not afford the private clinical investigations that they needed to have.

*I couldn’t afford to finance all the required further investigations privately and to buy all the drug types that I needed*. (user #12, 54-year-old female with NCD, facility B, Phase 1).

Almost all diabetes and hypertension care users attending the integrated clinic reported reduced requirements to purchase drugs from private pharmacies. This was observed during the second and third interviews.

The HCWs agreed that the integrated clinic had helped to make diabetic and hypertensive drugs available and affordable to the users through buffer stock supplies, although most expressed concerns about whether this situation would be sustainable.

*I am worried with the fact that what will happen when the project stops*, *would there be new files given or would there be drugs always availed*? *Hypertensive and diabetic patients had become so many and yet concentration [of resources] was always turned to HIV and immunization*. (HCW #3, a 49-year-old male, facility B, Phase 2).

As described earlier, the only exception was Facility A which operated a diabetes and hypertension patient club before establishing the integrated clinic, where they contributed money which helped to buy drugs in the event of a shortage.

#### Expenditure on self-care before establishment of the HIV/NCD integrated clinic

A small number of HCWs reported that diabetes and hypertension users were non-adherent to the treatment before the HIV/NCD clinic integration, an observation they attributed to poverty and an inadequate knowledge about their conditions.

*Stocked drugs are inadequate for the high number of hypertension patients*. *Even those for diabetes are not enough*. *Many of these patients cannot afford to buy from private pharmacies and they decide to go without medication till when the next supply comes*. (HCW #1, 38-year-old female, facility C, Phase 1).

However, it was not only a matter of not being able to afford drugs. Some HCWs reported that hypertensive users were less adherent and skipped drug refill appointments.

*I have a concern on how some patients don’t take their drugs religiously*. *Some patients miss their appointments and never show up*. *There were some hypertensive and diabetic patients who do not take these drugs [as being] very important and serious*. (HCW #7, a 51-year-old female, facility C, Phase 1).

Some HIV-care users reported being faced with a shortage of food, which sometimes affected their ability to adhere to treatment, because of drug side effects when they took their drugs on an empty stomach.

*I am faced with a challenge of inadequate food as I take my drugs daily*. *These drugs require a lot of food*, *and once I take drugs without food*, *I become dizzy and weak*. (user #28, 53-year-old female with NCD and HIV, facility B, Phase 1).

Given that the integrated clinic did not address food insecurity this situation did not change.

#### Expenditure on self-care after the establishment of the HIV/NCD integrated clinic

Challenges caused by poverty continued in the second and third phases of data collection. Indeed, there was an increasing number of HIV and diabetes care users who reported needing particular types of food. These people explained that this was because they were advised to revise their dietary intake by the HCWs, which some could not afford to do (because the foods they were advised to take were more expensive than their usual diet).

### Acceptability

#### Users views on the acceptability of integrated care before HIV/NCD care integration

Prior to the establishment of the integrated clinic, there were a small number of diabetes and hypertension service users who perceived that the integrated clinic could potentially expose them to tuberculosis (TB) infection, especially if the HIV users were ill with various opportunistic infections. These users expressed the view that if people living with HIV had linked to care in good time, they would not be sick, and therefore would not pose a risk.

*The only problem to the integration would be when HIV patients join while very sick with so many signs and symptoms [opportunistic infections] like TB [tuberculosis] A person with TB would definitely infect the other persons without the disease*. *TB patients cough incessantly*, *and others inhale the germs*. (user #12, 54-year-old female with NCD, facility B, Phase 3).

People with hypertension and diabetes did not openly express their stigma against people living with HIV sharing a clinic before integration occurred. Instead, their concerns focused on the risk to their own health from being close to people with contagious conditions.

#### HIV/NCD integrated clinic acceptability after the establishment of the integrated clinic

The majority of the HCWs reported that the users’ knowledge about their conditions improved with the establishment of the integrated clinic. The HCWs attributed the increased user knowledge to the intensified joint HIV/NCD patient education sessions.

*The patients’ awareness about their conditions has improved ever since the integrated clinic was established because health education is a must at the integrated clinic every time a patient comes for review or drugs refill*. *They (patients) are taught about what to eat*, *doing physical exercises*, *taking their pills religiously and cautioned against risk factors like alcohol*, *smoking*. (HCW #4, 28-year-old female, facility D, Phase 2).

This increased knowledge also helped users to understand conditions others attending the facility were accessing treatment for.

#### Impact of type of service on stigma after HIV/NCD care integration

Prior to HIV/NCD care integration, most of the people living with HIV accessing care reported that HIV-related stigma had greatly reduced in recent years. They further added that diabetes and hypertension were not stigmatised conditions.

*I don’t feel any form of stigma when I come to the hospital [facility] for HIV drugs because all those who come to collect the same drugs are like me*. *Even though one pinpointed [stigmatised]me*, *it does not make any sense nowadays because it is hard to know who is HIV infected and who is not*. (user #22, 26-year-old female with HIV, facility B, Phase 1).

Most HIV care users prior to the integration of HIV/NCD care explained that they enjoyed a good relationship amongst themselves which helped to minimise HIV related stigma.

*Sometimes I worry about my life but when I call one of my friends at the facility and they advise me*. *My friends at the facility usually tell me not to stress myself and I become strong*. (user #24, 23-year-old female with HIV, facility A, Phase 1).

No HCWs reported observing or expressing stigma towards the HIV and NCD care users prior to the establishment of the integrated clinic.

#### Acceptability of HIV/NCD integrated care after HIV/NCD care integration

Some users reported that the integrated clinic was acceptable because it had helped to further reduce stigma, as it was hard to tell what condition a person was being treated for. They explained that the file colour was identical for all conditions (under MOCCA) and that each participant consulted the clinician privately, so the person’s reason for seeking treatment was kept confidential. The HCW responses showed agreement with this view.

However, in one facility, facility C, registration, health education and dispensing occurred in the same room. With such an arrangement users could overhear what may be being said to an individual coming for their appointment or collecting their drugs.

Despite claims of reduced HIV related stigma and efforts to reduce it further, some young HIV care users were uncomfortable queuing for the integrated services amongst older people because of the age differences associated with the three conditions.

*There is a problem with the youth sitting with the older persons since the older persons might suspect the youths to be infected with HIV and this demoralises the youth when the older persons show it*. (user #24, 23-year-old female with HIV, facility A, Phase 3).

#### HCW-user relations before the HIV/NCD integrated clinic establishment

Most of the HIV and NCD users reported that they enjoyed good relationships with their HCWs prior to the establishment of the HIV/NCD integrated clinic. However, some users said that there were some HCWs who talked to them rudely and did not take time to explain things to them.

*We have some friendly health workers who take time to find out why you are missing your drug refill appointments and explain to you that missing one drug refill is detrimental to one’s health*. *There are some health workers who just talk to you in the presence of everyone about how you are doing badly*. (user #15, 59-year-old female with HIV, facility B, Phase 1).

#### HCW-patient relationship after the establishment of the HIV/NCD integrated clinic

Most of the users reported that improved HCW–patient relationships made the integrated clinic acceptable. They explained that HCWs who worked in the integrated clinic talked to them nicely and sometimes addressed them by name.

*The health workers at the integrated clinic treat all the users equally without discrimination*. *No one can know the other person’s condition*. *I feel peace getting treatment from the integrated clinic*. (user #20, 49-year-old female with HIV, facility A, Phase 3).

*There is a good relationship between me as a patient and the health worker*. *The medical team makes sure that they politely speak to you in a language that you best understand*, *any medical person will show you the love that you need in regard to the type of work s/he is doing*, *so that is what happens here*. (user #23, 50-year-old female with NCD, facility C, Phase 2).

The users did observe, however, that the HCWs were tough on those who missed their refill appointments.

HCWs reported that they were trying their best to deliver user friendly health services in the integrated clinic. They explained that they offered health education to the integrated clinic attendees, they explained the procedure to access care, and they did this in a friendly way. They added that all this was an effort to make the integrated clinic acceptable to the participants.

#### Efficiency of services of the HIV and NCD care before to HIV/NCD care integration

Almost all the users of the HIV and NCD care services reported that they appreciated the HCW and facility observation of the ‘first come, first serve’ principle prior to the HIV/NCD care integration. The users explained that it was important that they took turns to consult the HCW privately. HCW reported the same practice, and the importance of users taking turns.

*At this facility*, *the health workers are very tough on those who come later and want to be worked on first through skipping the queue*. *When one does that*, *the health workers can punish you by working on you last*. (user #13, 47-year-old female with HIV, facility C, Phase 1).

#### Efficiency of services after the establishment of the HIV/NCD integrated clinic

The users attributed the acceptability of the integrated HIV/NCD clinic to the continued observation of the `first come, first serve’ principle. The users explained that this continued practice, where someone who came later could not jump the queue, improved the acceptability of the integrated HIV/NCD clinics.

*Creating awareness through health education and secondly*, *we serve them as they come*, *we do first come first serve unless it is an emergency which we inform other patients about [the situation]*. (HCW #4, 28-year-old female, facility D, Phase 2).

During the second and third round of interviews, most of the diabetes and hypertension users reported that the integrated clinic was acceptable to them as drugs/diagnostics shortages had become rare and the service was more efficient.

*Whenever I come to pick my drugs*, *the health workers are available and ready to attend to all at all the serving points I go through till to the point of picking up the drugs*. *I have never faced any problem ever since I joined this facility*. (user #19, 32-year-old female with NCD, facility A, Phase 3).

As noted above, changes were beginning to creep in as MOCCA was coming to an end. Some drugs were beginning to run out.

*The quality hasn’t been bad*, *it is just that at times there are no drugs or if you are to get 2 types of drugs you will find that you have only gotten one type and the other type is not available yet you will have spent so much time waiting for drugs just to be told that the drugs are not available*. (user #17, 46-year-old male with NCD, facility C, Phase 3).

#### Length of waiting time before the HIV/NCD integrated clinic establishment

Most users reported that they spent a lot of time at the health facility each time they went for either a review or a drug refill prior to the establishment of the HIV/NCD integrated clinic. The users explained that most delay was at the consultation and the dispensing sections of the care pathway. The users further attributed the long waiting time to the fact that HCWs were faced with many patients to attend to. The majority of the HCWs agreed with this view.

*The number of patients attending the hypertension and diabetes clinic is so high yet we are few in number*. *The patients end up spending many hours at the facility*. (HCW #5, 54-year-old female, facility D, Phase 1).

Some users attributed the long waiting time to the fact that some HCWs reported late for work so there was a backlog of people to be seen throughout the day.

#### Length of waiting time after the establishment of the HIV/NCD integrated clinic

Most care users reported that with the introduction of the integrated clinic, their service waiting time had greatly reduced. They explained that users accessing care through the integrated clinic were often prioritised first when they went to the laboratory and at the dispensing window.

*I don’t feel delayed at all*. *The care providers always attend to me [and] worked on me the moment I set foot in the facility they also always give me a call reminding me to come to the facility a day prior to my appointment*. *It is something very unique about these healthcare providers*. *We follow the queue the same way we came in*. *if you came in earliest in the morning*, *then you leave earlier than any other patients*. (user #4, 60-year-old female with NCD and HIV, facility B, Phase 3).

#### Sitting arrangements before the HIV/NCD clinic integration

Almost all the users prior to the HIV/NCD integrated clinic establishment reported that their sitting arrangement was arranged by the HCWs, but within the designated area users were free to sit anywhere. They enjoyed sitting with their friends as they waited to be served. Among the things they discussed as they waited for the service were treatment side effects, adherence, dietary requirements, and service access challenges.

*There is no problem with the sitting arrangement*. *Everyone is free to sit anywhere with his/her friends and the queue to the consultation room is strictly observed*.(user #8, 59-year-old female with HIV, facility A, Phase 1).

#### Sitting arrangements after the establishment of the HIV/NCD integrated clinic

Most users reported that they were comfortable with the sitting arrangements under the integrated clinic. The users explained that although the HCWs organised these sitting arrangements, the people attending the facility were free to sit anywhere.

*I am comfortable […]*. *The chairs are enough and the place is not very crowded and when I come*, *I sit where I find a free seat*. (user #23, 39-year-old female with HIV, facility D, Phase 3).

The users explained that with the freedom to sit anywhere, they were still able to sit with their friends. The observations by the interviewers in the clinics before and after integration confirmed this explanation. While the waiting area was integrated, sitting patterns did not change markedly because people continued to sit with their friends. These friends were usually people seeking care for the same condition they had sat with before the clinic was integrated.

#### Number of visits to the clinic before HIV/NCD integration

Prior to the establishment of the integrated clinic, most users with multi-morbidities reported that they were making more frequent visits to the health facilities to seek care for a single condition per visit. The only exception was the users with multi-morbidities attending facility E where less severe hypertension and diabetes care needs were handled jointly with HIV. However, even from there the severe hypertension and diabetes care needs were referred to the nearby national referral hospital.

*People like me with more than one condition*, *must come to the facility on Tuesday for HIV and also come back on Thursday for hypertension within the same week*. *The clinic day for HIV is Tuesday while that of hypertension is Thursday*. *If you have both conditions*, *you visit the facility two times a week*. (user #3, 37-year-old female with HIV, facility D, Phase 1).

#### Number of visits after the HIV/NCD clinic integration

In the second and third phases of data collection, all the users reported that the integrated clinic was acceptable to them because it had indeed helped reduce their number of visits. This saved them transport money and time.

*One of the good things I have got from the integration is that*, *once someone is not doing fine*, *one gets free information on what to do*, *I get to know about the nutrition I am supposed to use for my better health*. *The integration has also reduced the amount of money and time that I use at the facility since I no longer spend any more money going to other facilities to pick drugs*. (user #29, 63-year-old male with NCD and HIV, facility A, Phase 3).

The HCWs reported that while having a single treatment visit was a good practice for users, it made more work for them.

*We treat patients with co-morbidities on one single visit because of the integration but there is a lot of paperwork*, *there is a lot of screening and taking off people’s blood always*, *taking pulse*, *filling cards*, *opening up files for clients*. (HCW #9, 34-year-old male, facility C, Phase 3).

Overall, the integrated clinic model resulted in services being accessible to HIV, hypertensive and diabetic users alike. While integration was blamed for an increased workload by some HCWs, on balance care users and healthcare providers welcomed the integrated model.

## Discussion

Our findings show that the integrated HIV, diabetic and hypertensive care delivery model improved diabetic and hypertensive care availability without compromising the provision of HIV care. Costs of transport were reduced for users, who were able to access care for more than one condition at a single visit. Users remarked that everyone looked the same in the waiting area, so in most clinics it was impossible to distinguish people seeking HIV care from those accessing care for diabetes or hypertension. We also found that integration helped to make hypertension and diabetes care more affordable by ensuring drugs were available for those conditions, and users did not have to meet prescription costs themselves.

In MOCCA the improvement in drug and diagnostics supply and availability was key to the acceptability of the integrated service. However, providing integrated care could not eradicate all the costs related to treatment, such as the costs of transport, and also food which users said they needed to support treatment adherence, a finding corroborated in other studies [26, 45, 46]. Out of pocket expenses have been shown to be a significant in impeding access to and retention in NCD care in Africa. A study in eastern Uganda among users with an NCD showed that over 92% of the diabetic users were unable to afford drugs purchases and this increased their rates of multi-morbidity [47]. Other research showed that NCD users in Uganda were found to be unable to afford the cost of care [48] and faced significant geographical constraints to accessing services [49, 50]. In South Africa, Malawi, Kenya and Uganda transport difficulties remained a key component not only in the integrated non-communicable diseases care but also for general healthcare access [7, 50–55]. We found in our study that even when the number of clinic visits was reduced, some people still struggled to meet the costs of transport to get to their appointments.

The challenges related to access, particularly costs, faced by users prior to MOCCA were not unusual in the region. There are multiple challenges in establishing integrated clinics–one stop shops–which provide both for people living with HIV, and people with an NCD who do not require HIV care: ensuring an NCD drug supply chain, establishing sustainable referral systems, ensuring adequately trained staff and providing monitoring across a range of different conditions [56–58]. In South Africa, a third of the hypertensive service users attending primary healthcare facilities could not access their prescribed drugs due to drug unavailability [59]. The provision of functional diagnostic equipment is needed to ensure that no users are asked to finance further investigations out of pocket or purchase drugs from private pharmacies [60]. Drug shortages and lack of diagnostic equipment negatively affected the service availability, with evidence from a number of studies showing that the provision of integrated care and management calls for a clear strategy to support a well organised health system in a supportive political environment for optimal outcomes [23, 48, 61–63]. As Kasaie, Weir [13] observe, for integration to be successful increased financial commitment is required to support and sustain services, particularly if they are to be delivered at scale. A review of healthcare policies in East Africa showed that in Uganda, Kenya, Tanzania and Rwanda, that while steps had been taken to change policy and practice to integrate NCD care into HIV care in the region, with some success, there were still challenges in sustaining integrated care [21].

Evidence from elsewhere has highlighted that a poor HCW-patient relationship, long queues, as well as stigma are barriers to integrated chronic disease care acceptability [23, 25, 50, 63, 64]. Our study findings on stigma corroborate those of research in South Africa where integrated care helped to reduce stigma among the people living with HIV [23, 50], although we found that this was not necessarily the case for younger people, who feared that they could be identified as someone with HIV because they thought they were too young to have an NCD.

Studies in rural Kenya and South Africa have reported that being dissatisfied with treatment was one of the reasons why hypertensive, diabetic and HIV service users skipped refill appointments [54, 61]. Ensuring quality care, is important to acceptability in any type of clinic. Where good care is provided, or where care is perceived to be of better quality to that provided prior to integration, users are less likely to be dissatisfied. A study in Malawi showed that NCD-HIV care integration increased NCD service users’ enrolment upon care integration resulting in similar retention levels for hypertension, diabetes and HIV as well as favourable clinical outcomes [65]. Other factors also play a part in maintaining access to care. Studies from rural Uganda and South Africa among hypertensive service users and HIV positive postpartum women respectively reported that having a supportive family and accessible health facilities promoted care acceptability [53, 55]; these are areas of support which go beyond the establishment of integrated clinics, but can be important in helping people stay in care.

### Strengths and limitations

The key strength of this study lies in the longitudinal design that allowed us to observe and document user and HCW perceptions and experiences of integrated clinics at different points. It is the first study of its kind in Uganda evaluating a one-stop clinic where people with any one or more of the chronic conditions (HIV, diabetes and hypertension) can attend. Another strength lies in our ability to triangulate perspectives of both those receiving integrated care and those providing care. We investigated the users and healthcare providers perceptions and experiences of an integrated care delivery model using multiple data sources which were triangulated across sources (users and providers) and methods (interview and observation). Other strengths include: the diverse range of health facility type and level and the different levels of integration at each of the facilities included in this study. Limitations centred on the difference across facilities in terms of the level and type of healthcare services offered and/or integration. Also, we recognise that MOCCA as a feasibility study provided drugs and diagnostic support which covered shortages in NCD drugs. We therefore do not know how available, affordable and acceptable the integrated HIV/NCD healthcare delivery model would be without this support.

## Conclusion

Our study shows that integrated healthcare services can save patients time, improve their access to services and increase their general health literacy. To be effective it also needs political commitment to primary healthcare; adequate and consistent drug, reagent and diagnostic equipment supplies, a fully supported healthcare workforce, and serious commitment to ensuring food security.

## Supporting information

S1 File Data collection tools (English and Luganda). (DOCX)

S1 Data Data table. (DOCX)
