# Supplementary material for: Integrated healthcare services for HIV, diabetes mellitus and hypertension in selected health facilities in Kampala and Wakiso districts, Uganda: A qualitative methods study
Source: PLOS Glob Public Health. 2022 Feb 3;2(2):e0000084. doi: 10.1371/journal.pgph.0000084 (PMC10021152; doi:10.1371/journal.pgph.0000084)
Supplement: S1 File — (DOCX) [file pgph.0000084.s001.docx]

**SUPPLEMENTARY FILE – study data collection tools**

**This file contains:**

In-depth interview guides for users for Phase 1, 2, 3 in English and Luganda

In-depth interview guides for Health Care Workers Phase 1, 2/3 in English only

Facility observation guide in English only

**IDI guide for users of the health facilities on integration – After Integration (Phase 1)**

Thank you for consenting to participate in this study.

As mentioned to you earlier, this is a collaborative research project involving Ministry of Health, Medical Research Council and TASO in Uganda; NIMR and Shree Hindu Mandal hospital in Tanzania; and the Liverpool School of Tropical Medicine in UK.

The aim of the research is to develop and evaluate a model of integrated HIV and Non Communicable Diseases care that is sustainable and leads to improved patients’ outcomes. Participation is purely voluntary and refusal does not affect the quality of care that you will receive.

We have selected some of you and your views will represent all other patients seeking NCD/HIV care at the clinics. The answers we get from you and several others will be analysed to get the general opinions on the feasibility of integrating HIV and NCD services at the point of care and in medicine refilling and distribution to the clients. We are kindly requesting you to be free and we assure you of the confidentiality of the information that you will provide. We request you to allow us use the tape recorder just to ensure the correctness of the information when writing the report.

Shall we proceed? Yes…….1 No……2

Date of interview…………………………

Participants ID……………………….

**Sociodemographic and treatment brief history**

1. Briefly tell me about yourself. Probe for age, sex, marital status, education level, income
2. What medical condition brough you to this facility today? (Probe: diabetes, hypertension, HIV, combination of conditions)
3. Briefly, tell me about your health seeking history in the last six months. Probe for asctiontaken before reaching to this facility, how long the participant has been accessing care from the facility, diabetes, hypertension and HIV treatment.

**Integration awareness**

1. Kindly tell me about the procedures that you have followed when you came to attend the clinic (probe from entry to the clinic, registration to drugs dispensing)?

How easy or difficult to comply with the stipulated procedures (probe about stigma, shyness, lack of clarity, etc.)?

1. We know that at this health facility there are different services provided to clients, and today you have visited with (DM, HT or HIV). Tell me about the quality of services that you have received today *Probe on*;
2. *interpersonal relationship,*
3. *waiting time,*
4. *distance to reach the clinic*
5. *costs (transport, health care services,*
6. *Availability of medicine and supplies etc.)*
7. Please tell me how comfortable were you with the sitting arrangement? Probe:
8. *Was the sitting arrangement by choice or directed?*
9. *Have you been able to know the condition of your neighbor that brought her/him to the health facility?*
10. *What did you discuss with your neighbor regarding your conditions and the services provided at this facility? How free the discussion was?*
11. As a person with (DM/HT/HIV), could you please tell me about the treatment (for your condition) that you receive from this facility (probe for duration (how long have you been to the service?), adherence among others, side effects, keeping of appointments etc.)
12. Please share your opinion on how the current management of DM/HT/HIV within this facility fits your needs as a patient) - this question should be tailored to the patient depending on his or her condition (s). (probe: screening, prescription, availability of prescribers and challenges faced in seeking care for his or her condition(s) among others)
13. Let us now talk about care and treatment for the three conditions (DM/HT/HIV) in one clinic, please share your experience of receiving care in the same clinic with other patients who have DM/HT/HIV: probe
14. *knowledge and understanding of the integration*
15. *What are your perceptions? (Probe of the willingness of the patients to continue seeking care in such integrated model, stigma, etc.)*
16. *Possible positives and negatives about integration e.g. time spent at the facility, perceived quality of care, follow up support etc.)*

**Ebinaalambika okunyumyamu n’abalwadde ababeera bava ku ddwaliro ng’obujjanjabi bugattibwa- n’oluvannyuma lw’okugattibwa. (Luganda)**

Webale kukkiriza kwetaba mu kunonyereeza kuno.

Nga bwewabuliddwa mu kusooka, okunonyereeza kuno kulimu ebitongole eby’enjawulo ng’eky’eby’obulamu, Medical Research Council, TASO mu Uganda; NIMR n’eddwaliro lya Hindu Mandal mu Tanzania; ne Ssetendekero wa Liverpool school of Tropical Medicine mu Bungereza.

Ekigendererwa ky’okunonyereeza kuno kwe kuzimba n’okwekkanya engeri ey’enzijjanjaba engatte ey’akawuka ka mukenenya n’edwadde ezitawona esoboka era egannyula omulwadde. Okwetaba mu kunonyereeza kuno kwa kyeyagalire era okugana kwo okwetaabamu tekikosa mutindo gwa bujjanjabi bw’ofuna.

Tulonzemu abamu ku mmwe era endowooza zammwe zijja kukiikirira endowooza z’abantu abalala abafuna obujjanjabi ku ndwadde ezitawona n’akawuka ka mukenenya ku kiliniki/ddwaliro lino. Byonooddamu ne byetufuna okuva eri abantu abalala abafuna obujjanjabi ku ndwadde ezitawona n’akawuka ka mukenenya ku kiliniki/ddwaliro lino bijja kwekennenyezebwa okufuna ekifaananyi ekyawamu ku kiki kyemulowooza ku nkola ey’okugatta obujjanjabi obw’endadde zino mu bifo awajjanjabirwa, webajjuliza eddagala n’okuligaba eri abalwadde. Tukusaba owulire eddembe era tukukakasa nti byonna by’onoddamu tujja kubikuuma nga bya kyama. Tukusaba okkirize tukozese akuuma kano akakwata amaloboozi okwongera okukakasa obutuufu bweby’onooba otuwadde nga tuwandiika alipoota.

what

Tugende mu maaso? Yee……..1 Needa……….2

Olunaku lw’okubuuzibwa/okuddamu ebibuuzo……………………………

Namba y’okunonyereeza ewebwa eyetabyemu

**Ebikwaata ku kikula n’ebyafaayo ebitono ku bujjanjabi.**

1. Mbulira katono nnyo ku bikukwatako. Buuza ku myaka, ekikula, embeera y’obufumbo, obuyigirize, ennyingiza y’eby’ensimbi.
2. Bulwadde ki obukuleese ku ddwaliro lino leero? ( Buuza ku; Sukaali, Puleesa, akawuka ka mukenenya oba ezissukka mwemu kwezo.)
3. Mbulira katono nnyo ku ngeri gy’ozze ofunamu obujjanjabi mu mwezi omukaaga egiyise. Buuza ku ki kyakola nga tannatuuka ku ddwaliro lino, ekiseera ky’amaze ng’afuna obujjanjabi okuva ku ddwaliro, Sukaali, Puleesa, n’obujjanjabi bw’akawuka ka mukenenya.

**Okumanya ebikwata ku nkola ey’okugatta obujjanjabi**

1. Mbuliira ku mitendera gy’ozze ogoberera ng’ojja ku kiliniki ( Buuza okuva webayingirira mu kiliniki, okwewandiisa ppaka webaagabira eddagaala)?

Kyangu oba kizibu kyenkana wa okugoberera emitendera egiba giikuweereddwa ( Buuza ku kusongebwamu ennwe, ensonyi, n’obutali bulambulukufu, n’ebirala).

1. Tukimanyi nti ku ddwaliro lino waliwo obuwereeza obw’enjawulo obuwebwa abalwadde naye leero ozze ne ( Sukaali, Puleesa oba akawuka ka mukenenya). Mbulira ku mutindo gw’obuweereza bwofunye wano leero. Buuza ku;
2. *Enkolagaana ey’obuntu*
3. *Ekiseera kyomala ng’olinda*
4. *Olugendo okutuuka ku ddwaliro*
5. *Ebisaale ( Ku ntabula, ku bujjanjabi)*
6. *Okubeerawo kw’eddagala n’engaba.*
7. Mbulira ku bumativu bwobadde owulira ku ngeri gyemutuulamu ? Buuza ku:
8. *Engeri gyemutuulamu ya kyeyagalire or esalibwawo?*
9. *Osobodde okumanya embeera / obulwadde obuviriddeko akulinaanye okujja ku ddwaliro?*
10. *Biki byewayogedde n’akuliranye ku mbeera/ndadde zo n’empereeza eziweebwa ku ddwaliro lino? Okwogera kubadde kwa ddembe kwenkana wa?*
11. Ng’omuntu alina (sukaali / puleesa / akawuka ka mukenenya), mbulira ku nzijjanjaba ( ey’embeera/obulwadde bwolina) gy’ofuna okuva ku ddwaliro lino ( buuza ku budde (ebbanga ly’amaze ng’afuna obujjanjabi?) obunyikivu n’ebiralala, ebizibu ebiva mu kumira eddagala, okukuuma ennaku ezikuweeredwa eddwaliro n’ebirala.)
12. Mbulira ku kyolowooza ku ngeri endabirira ya sukaali / puleesa / akawuka ka mukenenya oba etuukana obwetaavu bwo ng’omulwade mu ddwaliro muno – ekibuuzo kino kirina kubuuzibwa omulwadde okuzinzira ku mbeera/obulwadde bwe. (Buuza ku webaakeberera ani mulwadde natali, webawandiikira obujjanjabi omuntu bw’afuna, okubeerawo kw’abawandiika obujjanjabi n’obuzibu bwebasanga nga banonya obujjanjabi ku mbeera zabwe n’ebirala.
13. Katwogere ku bujjanjabi n’endabirira ku ndwadde zino esaatu (Sukaali / Puleesa / Akawuka ka mukenenya) mu kiliniki emu. Mbulira ku byoyitamu mu kufuna obujjanjabi mu kiliniki emu n’abalwadde abalala abalina Sukaali / Puleesa / Akawuka ka mukenenya; Buuza ku;
14. Okumanya n’okutegeera ku nkola ey’okugatta *(obujjanjabi*.) added to maintain the intended meaning of okugatta in this regard.)
15. Olina ndowooza ki? ( buuza ku kwagala kw’abalwadde okweyongera okunonya obujjanjabi wansi w’enkola ey’okugatta, okusongebwamu ennwe n’ebirala)

Ebirungi n’ebibi ku nkola ey’okugatta obujjanjabi okugeza ng’obudde bwebamala ku ddwaliro, endowooza ku mutindo gw’obujjanjabi, obuyambi bw’okulondola abalwadde n’ebirala)

**IDI guide for users of the health facilities on integration – After Integration (Phase 2)**

Thank you for consenting to participate in this study.

As mentioned to you earlier, this is a collaborative research project involving Ministry of Health, Medical Research Council and TASO in Uganda; NIMR and Shree Hindu Mandal hospital in Tanzania; and the Liverpool School of Tropical Medicine in UK.

The aim of the research is to develop and evaluate a model of integrated HIV and Non Communicable Diseases care that is sustainable and leads to improved patients’ outcomes. As you may recall, you were interviewed before the implementation of this project and now we want to get your views as you continue receiving care in the integrated clinic. Participation is purely voluntary and refusal does not affect the quality of care that you will receive.

We selected some of the clients from the first phase and we will continue engaging them in the subsequent phases, so so your views will represent all other patients seeking NCD/HIV care at the integrated clinic. The answers we get from you and several others will be analyzed to get the general opinions on how service delivery progresses for more improvement as we continue with implementation. We are kindly requesting you to be free and we assure confidentiality of the information that you will provide. We request you to allow us use the tape recorder just to ensure the correctness of the information when writing the report.

Shall we proceed?

Yes…….1 No……2

Date of interview…………………………

Participants ID……………………….

**Sociodemographic and treatment brief history**

1. Briefly tell me about yourself. Probe for age, marital status, education level, income
2. What type of medical condition brought you to this facility today (probe: diabetes, hypertension, HIV, combination of conditions)
3. Briefly, tell me about your health seeking history since the integrated clinic was established. Probe for how long the participant has been accessing care from this clinic for either diabetes, hypertension and HIV treatment.
4. Kindly tell me about the procedures that you followed when you came to attend the integrated clinic (probe from entry to the clinic, registration to drugs dispensing)?

How easy or difficult to comply with the stipulated procedures (probe about stigma, shyness, lack of clarity, etc.)?

1. We know that at this integrated clinic there are different services provided to clients, and today you have visited with (DM, HT or HIV or both ). Tell me about the quality of services that you have received today *Probe on*;
2. *interpersonal relationship (probe for language, services whether friendly or not, advises provided etc),*
3. *waiting time,*
4. *distance to reach the clinic/accessibility,*
5. *costs (transport, health care services)*
6. *Availability of medicine and supplies etc.)*
7. Please tell me how comfortable were you with the sitting arrangement in the integrated clinic? Probe:
8. *Was the sitting arrangement by choice or directed? Kindly explain the level of satisfaction with the sitting arrangement*
9. *Have you been able to know the condition of your neighbor that brought her/him to the integrated clinic?*
10. *What did you discuss with your neighbor regarding your conditions and the services provided at this clinic? (if no discussion was made ask why). How free the discussion was?*
11. As a person with (DM/HT/HIV), could you please tell me about the treatment (for your condition) that you receive from this integrated clinic (probe for duration (how long have you been to the integrated service?), adherence among others, side effects, keeping of appointments etc.)
12. Please share your opinion on how the current management of DM/HT/HIV within integrated clinic fits your needs as a patient) - this question should be tailored to the patient depending on his or her condition (s). (probe: screening, prescription, availability of prescribers and challenges faced in seeking care for his or her condition(s) among others)
13. Let us now talk about care and treatment for the three conditions (DM/HT/HIV) in one/integrated clinic, please share your views of receiving care in the same clinic with other patients who have DM/HT/HIV: probe
14. *Knowledge and understanding of the integration*
15. *What are your perceptions? (Probe of the willingness of the patients to continue seeking care in such integrated model, stigma, etc.)*
16. *Possible positives and negatives about integration e.g. time spent at the clinic, perceived quality of care, follow up support etc.)*

**Ebinalambika okunyumyamu n’abalwadde ababeera bava ku ddwaliro ng’obujjanjabi bugattibwa- Omutendera ogw’okubbiri (Luganda)**

Webale kukkiriza kwetaba mu kunonyereza kuno.

Nga bwe wabuliddwa mu kusooka, okunonyereza kuno kulimu ebitongole eby’enjawulo nga ekyo by’obulamu, Medical Research Council, TASO mu Uganda; NIMR n’eddwaliro lya Hindu Mandal mu Tanzania ne Ssetendekero wa Liverpool school of Tropical Medicine mu Bungereza.

Ekigendererwa ky’okunonyereza kuno kwe kuzimba n’okwekanya enzijjanjaba ey’awamu ku akawuka kamukenenya n’endwadde ezitawona esoboka era eganyula omulwadde. Nga bwojjukira emabegako awo, twayogerako nawe nga enkola eno egata obujjanjabi bw’akawuka kamukenenya n’endwadde ezitawona tenaba kutekebwa mu nkola. Kati twagara okufuna endowozayo nga bwe weyongera okufuna obujjanjjabi ku kiliniki ejjanjaba mu nkola ey’okugatta obujjanjabi bwa kawuka kamukenenya n’endwadde ezitawona. Okwetaba mu kunonyereza kuno kwa kyeyagalire era okugana kwo okwetabamu tekikosa mutindo gwa bujjanjabi bw’ofuna.

Twalondomu abamu ku mmwe abaali betabye mu mukunoyereza kuno nga kutandika era tujja kwongera okwogeraganya nabo mu mitendera emilala egy’ okunonyereza kuno gye bugya mu maaso. Era endowozayo ejja kukiikirira ez’abantu abalala abafuna obujjanjabi ku kiliniki eno ejjanjaba mu nkola ey’okugata obujjanjabi obwe ndwadde ezitawona n’akawuka ka mukenenya. By’ onoddamu ne bye tunafuna okuva eri abantu abalala bigya kwe kenenyezebwa okufuna ekifaananyi eky’awamu ku ngeri omutindo gw’obujjanjabi gye guyinza okutumbulwa nga bwe tweyongera okugata obujjanjabi bwa kawuka kamukenenya ne ndwadde ezitawona.

Tukusaba owulire eddembe era tukukakasa nti byonna by’onoddamu tujja kubikuuma nga bya kyama. Tukusaba okirize tukozese akuuma kano akakwata amaloboozi okwongera okukakasa obutuufu bwa by’onoba otuwadde nga tuwandiika alipoota.

Tugende mu maaso?

Yee……..1 Needa……….2

Olunaku lw’okubuzibwa/okuddamu ebibuuzo……………………………………

Namba y’okunonnyereza ewebwa eyetabyemu……………………………………

**Socio demographic information.**

1. Mubufunze, mbulira ku bikukwatako. Buuza; yazalibwa ddi, mufumbo, obuyigirize ne by’ akola okufuna ensimbi.
2. Bulwaddeki obukulese ku ddwaliro lino leero? ( Buuza ku; Sukaali, Puleesa, akawuka ka mukenenya oba ezisukka mw’emu kuzino)

**Brief treatment history**

1. Mubufunze, mbulira ku ngeri gy’ozze ofuna obujjanjabi okuva enkola oy’okugata obujjanjabi bw’akawuka kamukenenya n’ endwadde ezitawona mu kiliniki emu lweyatekebwawo. Buuza ebanga ly’ amaze ng’afuna obujjanjabi okuva ku kiliki ey’ awamu ku ndwadde ya ? Obujjanjabi afuna bwa bulwaddeki? Sukaali/Puleesa oba akawuka kamukenenya.

Mbuliramuko, ku mitendera gy’ ogoberera nga oze ku kiliniki/ddwaliro lino, erilina enkola ey’okugata obujjanjabi bw’akawuka kamukenenya n’endwadde ezitawona. ( Buuza okuva weba yingirira mu kiliniki/ddwaliro, webe wandiisiza ppaka weba gabira eddagala).

Kyangu oba kizibu kyenkana wa okugoberera emitendera egiba gikuwereddwa ( Buuza ku kusongebwamu ennwe, ensonyi, obutali bulambulukufu, n’ebilala)?

1. Tukimanyi nti ku kiliniki/ddwaliro lino eriyina enkola oy’okugata obujjanjabi bw’akawuka kamukeneya n’endwadde ezitawona (e ly’awamu) waliwo obuwereza obwenjawulo obuwebwa obalwadde era leer’ oze ofune empereza ku bulwadde bwa (Sukaali, Puleesa , akawuka ka mukenenya oba ebiri ku zino). Kati mbulira ku mutindo gw’ obuwereza bw’ofunye leero. Buuza ku;
2. *Enkolagaana ey’obuntu (buzza ku lulimi olukozesebwa, empereza ewebwa n’omukwano oba nneda okuwebw’amagezi n’ebilala)*
3. *Ekiseera kyomala nga olinze*
4. *Olugendo okutuuka kukiliniki /etukibwako /i lutambulikika*
5. *Ebisale ( Ku ntambula, ku bujjanjabi)*
6. *Okuberawo kw’eddagala n’ebikola ebilala.*
7. Mbulira ku bumativu bw’obadde owulira ku ngeri gye mutulamu mu ku kiliniki/ddwaliro ey’awamu egaba obujjanjabi obw’awamu eri akawuka kamukenenya ne ndwadd’ezitawona? Buuza ku:
8. *Engeri gye mutula ya kyeyagalire oba yabuwaze? Nyonyola ku bumativu bwo n’engeri gye mutuula.*
9. *Osobodde okumanya embera / obulwadde obuviriddeko akulinaanye okujja ku kiliniki eya’wamu?*
10. *Biki bye mwanyumiza n’akuliranye ku mbeerayo / n’empereza egabibwa ku kiliniki eno ? ( Bwe kiba nti tebanyumiza, buuza lwaki ) Okunyumyamu kwabadde kwa ddembe kwenkana wa?*
11. Ng’omuntu alina (sukaali / puleesa / akawuka ka mukenenya), mbulira ku nzijjanjaba ( ey’embera/obulwadde bw’ olina) gy’ofuna okuva ku kiliniki eno eya’wamu . (Buuza ku kiseera( ebbanga ly’amaze ng’afuna obujjanjabi okuva mu kiliniki eya’wamu ) obunyikivu mu kumira eddagala, obuvune , okukuuma ennaku ezikuweredwa eddwaliro n’ebirala.
12. Mbulira ku ky’olowoza ku ngeri endabirira ya sukaali / puleesa / akawuka ka mukenenya bw’ etuukana n’obwetavu bwo ng’omulwade. **This question should be tailored to the patient depending on his or her condition (s).** (Buuza ku webakeberera ani mulwadde natali, webawandiikira obujjanjabi omuntu bw’anafuna, okubeerawo kw’abawandiika obujjanjabi n’obuzibu bwe basanga nga banonya obujjanjabi ku mbera zabwe n’ebirala.

9.Katwogere ku bujjanjabi n’endabirira ku ndwadde zino esaatu (Sukaali / Puleesa / Akawuka ka mukenenya) mu kiliniki emu. Mbulira ku by’oyitamu mu kufuna obujjanjabi mu kiliniki emu n’abalwadde abalala abalina Sukaali / Puleesa / Akawuka ka mukenenya; Buuza ku;

1. Okumanya n’okutegeera ku nkola ey’okugata obujjanjabi bwe ndwadde zino
2. Endowooza yo eli etya ? ( Buuza ku bumalilivu bwa’balwade okweyongera okufuna obujjanjabi wansi w’enkola ey’okugata, okusongebwamu ennwe n’ebirala)

c) Ebirungi n’ebibi ku nkola ey’okugata okugeza; Nga 0budde bwe bamala ku kiliniki endowooza ku mutindo gw’obujjanjabi, obuyambi bw’okulondola abalwadde n’ebirala)

**IDI guide for users of the health facilities on integration – After Integration (Phase 3)**

Thank you for consenting to participate in this study.

As mentioned to you earlier, this is a collaborative research project involving Ministry of Health, Medical Research Council and TASO in Uganda; NIMR and Shree Hindu Mandal hospital in Tanzania; and the Liverpool School of Tropical Medicine in UK.

The aim of the research is to develop and evaluate a model of integrated HIV and Non Communicable Diseases care that is sustainable and leads to improved patients’ outcomes. As you may recall, you were interviewed before the implementation of this project and now we want to get your views as you continue receiving care in the integrated clinic. Participation is purely voluntary and refusal does not affect the quality of care that you will receive.

We selected some of you and got interviewed in the first and second phases and we would like to interview you for the third-which is the final phase of this feasibility study. Your views will represent all other patients seeking NCD/HIV care at the integrated clinic. The answers we get from you and several others will be analyzed to get the general opinions on how service delivery progresses for more improvement as we continue with implementation. We are kindly requesting you to be free and we assure confidentiality of the information that you will provide. We request you to allow us use the tape recorder just to ensure the correctness of the information when writing the report.

Shall we proceed?

Yes…….1 No……2

Date of interview…………………………

Participants ID……………………….

**Sociodemographic and treatment brief history**

1. Briefly tell me about yourself. Probe for age, marital status, education level, income
2. What type of medical condition brought you to this facility today (probe: diabetes, hypertension, HIV, combination of conditions)
3. Briefly, tell me about your health seeking history since the integrated clinic was established. Probe for how long the participant has been accessing care from this clinic for either diabetes, hypertension and HIV treatment.
4. Kindly tell me about the procedures that you followed when you came to attend the integrated clinic (probe from entry to the clinic, registration to drugs dispensing)?

How easy or difficult to comply with the stipulated procedures (probe about stigma, shyness, lack of clarity, etc.)?

1. We know that at this integrated clinic there are different services provided to clients, and today you have visited with (DM, HT or HIV or both ). Tell me about the quality of services that you have received today *Probe on*;
2. *interpersonal relationship (probe for language, services whether friendly or not, advises provided etc),*
3. *waiting time,*
4. *distance to reach the clinic/accessibility,*
5. *costs (transport, health care services)*
6. *Availability of medicine and supplies etc.)*
7. Please tell me how comfortable were you with the sitting arrangement in the integrated clinic? Probe:
8. *Was the sitting arrangement by choice or directed? Kindly explain the level of satisfaction with the sitting arrangement*
9. *Have you been able to know the condition of your neighbor that brought her/him to the integrated clinic and how? If no kindly explain*
10. *What did you discuss with your neighbor regarding your conditions and the services provided at this clinic? (if no discussion was made ask why). How free the discussion was?*
11. As a person with (DM/HT/HIV), could you please tell me about the treatment (for your condition) that you receive from this integrated clinic (probe on adherence among others, side effects, keeping of appointments etc.)
12. Please share your opinion on how the current management of DM/HT/HIV within integrated clinic fits your needs as a patient) - this question should be tailored to the patient depending on his or her condition (s). (probe: screening, prescription, availability of prescribers and challenges faced in seeking care for his or her condition(s) among others)
13. As a client who has attended multiple visits in this integrated clinic, please share your views of receiving care in the same clinic with other patients who have DM/HT/HIV: probe
14. *Knowledge and understanding of the integration*
15. *What are your perceptions? (Probe of the willingness of the patients to continue seeking care in such integrated model, stigma, etc.)*
16. *Possible positives and negatives about integration e.g. time spent at the clinic, perceived quality of care, follow up support etc.)*
17. We are now coming to an end of the integrated clinic sessions. If you are given an option to choose whether to continue with the integrated clinic or to go back to the normal routine (i.e. diabetes, hypertension or HIV clinic) which are vertical/stand alone, what would be your preference? Probe for the reasons

Probe: if she/he can recommend to other clients to attend integrated clinic in future

1. What do you think could have been done better to improve service delivery in the integrated clinic?

THANK YOU

**Ebinalambika Okunyumyamu n’abalwadde ababeera bava ku ddwaliro elyo’obujjanjabi obugattibwa: Omutendera ogw’okussatu (Luganda)**

Webale kukkiriza kwetaba mu kunonyereza kuno.

Nga bwe wabuliddwa mu kusooka, okunonyereza kuno kulimu ebitongole eby’enjawulo nga ekyo by’obulamu, Medical Research Council, TASO mu Uganda; NIMR n’eddwaliro lya Hindu Mandal mu Tanzania ne Ssetendekero wa Liverpool school of Tropical Medicine mu Bungereza.

Ekigendererwa ky’okunonyereza kuno kwe kuzimba n’okwekanya enzijjanjaba egat’awamu akawuka kamukenenya n’endwadde ezitawona esoboka era eganyurwa omulwadde. Nga bwojjukira emabegako’awo, twayogerako nawe nga enkola eno egata obujjanjabi bw’akawuka kamukenenya n’endwadde ezitawona tenaba kutekebwa mu nkola. Kati twagara okufuna endowozayo nga bwe yongera okufuna obujjanjjabi ku kiliniki ejjanjaba mu nkola ey’okugatta obujjanjabi bwa kawuka kamukenenya n’endwadde ezitawona. Okwetaba mu kunonyereza kuno kwa kyeyagalire era okugana kwo okwetabamu tekikosa mutindo gwa bujjanjabi bw’ofuna.

Twalondomu abamu ku mmwe, ella, netunyumyamu namwe ku’mutendella ogwasooka, nogwokubilli, nante, ella twagalla tunyumyemu namwe ku’mutendella ogwo’okusatu, ella omutendella ogusembayo mu’musoma gunno. Endowozayo ejja kukiikirira z’abantu abalala abafuna obujjanjabi ku kiliniki eno ejjanjaba mu nkola ey’okugata obujjanjabi bwe ndwadde ezitawona n’akawuka ka mukenenya. By’ onoddamu ne bye tunafuna okuva eri abantu abalala abafuna bijja kwe kenenyezebwa okufuna ekifaananyi eky’ awamu ku kiki kye mulowoza ku’mepereza eno gye tambulamu nga bwe tugenda mu maaso n’enkola ey’okugata awamu obujjanjabi bwa kawuka kamukenenya ne ndwadde ezitawona. Tukusaba owulire eddembe era tukukakasa nti byonna by’onoddamu tujja kubikuuma nga bya kyama. Tukusaba okirize tukozese akuuma kano akakwata amaloboozi okwongera okukakasa obutuufu bwe by’onoba otuwadde nga tuwandiika alipoota.

Tugende mu maaso?

Yee……..1 Needa……….2

Olunaku lw’okubuuzibwa/okuddamu ebibuuzo……………………………

**Namba yo’kunonnyereza ewebwa eyetabyemu**…………………………

1. **Ebikwata ku yetabye mu kunonyeleza, nebyafayo bye mukufunna obbnjajjabi.** Mubufunze, mbulira ku bikukwatako. Buuza; yazalibwa ddi, mufumbo, by’akola okufuna ensimbi, obuyigirize bw’ayina.
2. Bulwaddeki obukulese ku ddwaliro lino leero? ( Buuza ku; Sukaali, Puleesa, akawuka ka mukenenya oba ezisukka mw’emu kwezo)
3. Mubufunze, mbulira ku ngeri gy’ozze ofunamu obujjanjabi okuva enkola oy’okugata obujjanjabi bw’akawuka kamukenenya nendwadde ezitawona mu kiliki emu. Buuza amaze bangaki ng’afuna obujjanjabi okuva ku kiliki eno elina enkola ey’okugata obujjanjabi awamu obwa Sukaali, Puleesa, akawuka ka mukenenya?
4. Mbuliramuko, ku mitendera gy’ ogoberera nga oze ku kiliniki/ddwaliro lino, erilina enkola ey’okugata obujjanjabi bw’akawuka kamukenenya n’endwadde ezitawona. ( Buuza okuva weba yingirira mu kiliniki/ddwaliro, webe wandiisiza ppaka weba gabira eddagala)? Kyangu oba kizibu kyenkana wa okugoberera emitendera egiba gikuwereddwa ( Buuza ku kusongebwamu ennwe, ensonyi, n’obutali bulambulukufu, n’ebilala).
5. Tukimanyi nti ku ddwaliro lino erilina enkola ey’okugata obujjanjabi bw’akawuka kamukenenya n’endwadd’ ezitawona, waliwo empereza ez’njawulo eziwebwa abalwadde. Olwa leero’ ozze, ofune empereza ku bulwadde bwa; Sukaali, Puleesa oba akawuka ka mukenenya). Mbulira ku mutindo gw’ empereza bw’ofunye leero. Buuza ku;
6. *Enkolagaana ey’obuntu (buzza ku lulimi olukozesebwa, empereza ewebwa n’omukwano, okubulirirwa etc)*
7. *Ekiseera/obudde kyomala nga olinze*
8. *Olugendo okutuuka ku ddwaliro/kiliki lutambulikika*
9. *Ebisale ( Ku ntambula, ku bujjanjabi)*
10. *Okuberawo kw’eddagala n’ebikola ebilala.*
11. Mbulira ku bumativu bw’obadde owulira ku ngeri gye mutulamu mu ddwaliro/kiliki egaba obujjanjabi obw’awamu eri akawuka kamukenenya ne ndwadd’ezitawona? Buuza ku:
12. *Engeri gye mutulamu ya kyeyagalire oba yabuwaze? (elagibwa bulagibwa/esalibwawo abalala). Yogera ku bumativu bw’olina n’engeri gye mutuulamu ku ddwaliro/kiliki eno*
13. *Osobodde okumanya embera / obulwadde obuviriddeko akulinaanye okujja ku ddwaliro? Wobba tosobodde, kiki ekikuziyiza*
14. *Biki bye wayogedde n’akuliranye ku mbeera/ndwaddezo n’empereza eziwebwa ku ddwaliro lino? Okwogera kubadde kwa ddembe kwenkana wa? Singa tewabaddewo, kwogeranya kwonna, lwaki? Okwogera kwabadde kwadembe?*
15. Ng’omuntu alina (sukaali / puleesa / akawuka ka mukenenya), mbulira ku nzijjanjaba ( ey’embera/obulwadde bw’ olina gy’ofuna okuva ku ddwaliro lino erilina nkola ey’okugata obujjanjabi. (Buuza ku bunyinkiviu bwo’kumilla edagalla, obuzivu obuva mukumilla edagala, okukuma enaku ezokuffuna edagala etc.)
16. Mbulira ku ky’olowoza ku ngeri endabirira ya sukaali / puleesa / akawuka ka mukenenya bw’ etuukana n’obwetavu bwo ng’omulwade. this question should be tailored to the patient depending on his or her condition (s). (Buuza ku webakeberera ani mulwadde natali, webawandiikira obujjanjabi omuntu bw’anafuna, okubeerawo kw’abawandiika obujjanjabi n’obuzibu bwe basanga nga banonya obujjanjabi ku mbera zabwe n’ebirala.
17. Nga aze affuna obujjanjabi munaku ezitali zimu ku kilinki enjjanjaba ndwadde zino esaatu (Sukaali / Puleesa / Akawuka ka mukenenya) mu kiliniki emu. Mbulira ku by’oyitamu mu kufuna obujjanjabi mu kiliniki emu n’abalwadde abalala abalina Sukaali / Puleesa / Akawuka ka mukenenya; Buuza ku;
18. *Okumanya n’okutegeera ku nkola ey’okugata obujjanjabi bw’endwadde zino*
19. *Olina ndowoza ki? ( buuza ku kwagala kw’abalwadde okweyongera okufuna obujjanjabi bw’endwadde zino wansi w’enkola ey’okubugata, okusongebwamu ennwe n’ebirala)*
20. *Ebirungi n’ebibi ku nkola ey’okugata obujjanjabi bw’okugeza ng’obudde bwe bamala ku ddwaliro, endowooza ku mutindo gw’obujjanjabi, obuyambi bw’okulondola abalwadde n’ebirala*
21. Kati, nga tufundikilla enkola yo’kufunna obujanjabi mu kilinki eyawamu. Wo’bba owelwebwa okulondawo, okusigala mu kilinki eyawamu, no’kuffuna obunjanjabi nga’wekibadde kikolebwa emmabegga ( i.e sukali, puleesa, obba akawuka) nga obujanjabi ofunna bwa ndwade emu yokka buli’lwobozze ku kilinki. Wandibadde olondawo ki? Ella, Mbulila ensonga zo. Buzza: Obba anakubilliza abalalwadde abbalala okwetanilla kilinki eyawamu gyebujjya
22. Olowoza kiki ekyandikolebwa okulongosa, no’kutumbulla empelezza ya kilinki engate eyawamu?

Webbale

**IDI guide for health facility workers (at NCD and HIV clinic/OPD clinician) (Phase 1) (English only)**

My name is ….........................I am working with the National Institute for Medical Research (Tanzania)/Medical Research Council/Uganda Virus Research Institute (MRC/UVRI). This is a collaborative project between NIMR, Hindu-Mandal Hospital, TASO Uganda, Medical Research Council/Uganda Virus Research Institute and Liverpool School of Tropical Medicine. In this study we are interested in getting opinions on the feasibility of integrating HV and NCD care services at the service delivery point. We have selected some few health facility workers who work closely with patients seeking NCD/HIV care at the clinics. The answers we get from you and several others will be analysed to get the general opinions on the feasibility and acceptance of integrating HIV and NCD services at the point of care. We are kindly requesting you to take part, be free and we assure you of the confidentiality of the information that you will provide. We request you to allow me use the tape recorder just to ensure the correctness of the information when writing the report.

Shall we proceed ? Yes----------------No………………….

1. Tell me about yourself. Probe for; age, sex, marital status, ethnic group, occupation and educational level, title/position, roles at the facility.
2. What chronic diseases do you treat at this facility? Probe for; a). HIV, diabetes and hypertension. b) how big the burden for each of these and co-morbidity. c) what explains this burden of HIV, diabetes and hypertension? d) If you compare number of clients seeking health care services for HIV/NCD, what proportions of these do you think have co-morbidity? (Either HIV/hypertension, HIV/diabetes, hypertension diabetes, At HIV/hypertension/diabetes?) and why
3. What services do you provide at this facility for HIV, diabetes and hypertension? NB ask one condition at a time. (probe Diagnostic, medication, consultation, health education sessions or all etc)
4. At an individual level, what do you to ensure HIV, DM and HT care is user friendly? Probe for patient-health worker relationship, patient-patient relationship

(b) At an institutional level, what does the facility management do to ensure HIV, DM and HT care is user friendly, Probe for; diagnostic, drugs availability, health worker availability, waiting area and time, sitting arrangements, health education sessions, encouraging patient-patient’ relationship/associations

1. In the past 6months, what changes have been implemented in the HIV, DM and HT care provision? Probe for; (a)integration, what has been integrated (if not mentioned probe for the possibility of integration)

What services do you think should be integrated? Probe for; diagnostic, medication, consultation, health education or all et and the reasons

Benefits of the HIV, DM and HT care integration

(b) Challenges of the HIV, DM and HT care integration

1. What do you think is the better way of integration (either partial or full integration)? Probe for the reasons.
2. Probe; how feasible for such integrated model either partial or full. i.e how ready is the clinic/facility in terms of number of trained health workers available, space/environment, diagnostic facilities, medicine etc
3. How satisfied will you be with such integrated model? Probe for the reasons
4. Based on your experiences with the clients/patients in this clinic, what do you think need to be done differently to ensure user friendly services in the integrated clinic? Probe for;
5. At an individual level; patient-health worker relationship, patient-patient relationship
6. At an institutional level, in terms of ensuring diagnostic equipment, drugs, health worker availability, improving waiting area and time, sitting arrangements, health education sessions, encouraging patient-patient’ relationships/associations
7. Based on the experience of working with the clients, what will be their attitude and perception towards such integrated model? Probe on changes in health seeking behavior for DM, HT ad HIV patients specifically on access, retention and adherence to treatment

What do you think should be done to ensure that they (clients/patients) ensure that they develop positive attitude towards the integration?

**IDI guide for health facility workers (at NCD and HIV clinic/OPD clinician), Phase II/III (English only)**

My name is ….........................I am working with the National Institute for Medical Research (Tanzania)/Medical Research Council/Uganda Virus Research Institute (MRC/UVRI). This is a collaborative project between NIMR, Hindu-Mandal Hospital, TASO Uganda, Medical Research Council/Uganda Virus Research Institute and Liverpool School of Tropical Medicine. As you are aware of, the Ministry of Health has already started to pilot the feasibility of integrating HIV, hypertension and diabetes services. We are therefore interested to get your opinions and experiences of this model following its implementation. We initially selected some few health facility workers who worked closely with patients seeking NCD/HIV care at the clinics in the baseline and midline assessment, and now we are under the third/phase as we wind up this feasibility study. The answers we get from you and several others will be analysed to get the general opinions on the performance and acceptance of integrating HIV and NCD services at the point of care. We are kindly requesting you to take part, be free and we assure you of the confidentiality of the information that you will provide. We request you to allow me use the tape recorder just to ensure the correctness of the information when writing the report.

1. First of all, I would like to get the information about your age, sex, marital status, ethnic group, occupation, educational level, title/position, roles at this facility/clinic
2. What chronic diseases do you treat at this integrated clinic? Probe for; a). HIV, diabetes and hypertension.

- How big is the burden for each of these disease conditions, and what explains this burden of HIV, diabetes and hypertension?
- If you compare number of clients seeking health care services for HIV/NCD, what proportions of these do you think have co-morbidity? (Either HIV/hypertension, HIV/diabetes, hypertension/diabetes, HIV/hypertension/diabetes?) and why

1. At this clinic, what modality of integration is implemented (partial or full?) and why

- Probe for services provided at this integrated clinic NB ask one condition at a time. (probe Diagnostic, medication, consultation, health education sessions or all etc)

What is your general experience in managing patients in the integrated clinic? (probe for strength, weaknesses, threats and opportunities)

1. What benefits have you noted for HIV, DM and HT care integration?
2. What challenges have you noted on the HIV, DM and HT care integration?
3. How satisfied are you with such integrated model? Probe for the reasons
4. Using your experience of integration, what do you think is the better way of integration (either partial or full integration)? Probe for the reasons
5. Based on the experience of working with the clients in this integrated clinic, what is their attitude and perception towards such integrated model? (Probe on changes on seeking behavior for DM, HT and HIV patients specifically on access, retention and adherence to treatment?

What do you think should be done to ensure that they develop positive attitude towards the integration?

1. As you might be aware that we are approaching to an end of the integrated clinic for the enrolled clients. Based on your experience with the clients, if they are given an option to choose either to continue receiving care in the integrated clinic or go back to their routine/vertical and standalone clinic, what do you think will be their choice and why?
2. As a care provider, you have managed combination of these disease conditions (HIV, DM, HT) in the same clinic for several months. If you are also asked to choose whether to continue with such integrated model in this facility or not, what would be your option and why?

Probe: what is your willingness/readiness to continue providing integrated care? Kindly explain

1. What do you think could have been done better to improve service provision in the integrated clinic?

**THANK YOU**

**Facility observation guide (English only)**

The aim of the facility observation guide is to document how services are provided in each health facility before integration. This is an addition to the facility assessment tool and is more descriptive of the physical infrastructure, its location as well as any other observations on how care is delivered or how patients behave in the clinic. This guide is to help ensure that we observe the model of non-communicable disease and HIV care provided both pre and post integration in a consistent manner across the sites. This should be completed by the study co-ordinator or medical officer or a member of the study team based at the facility (section one) and a social scientist (section two).

Please use free text and add any other observations at the end.

**SECTION ONE**

| 1. **Description of physical infrastructure** | |
| --- | --- |
| - 1. Provide photographs or a floor plan |  |
| - 1. What level of health facility is this? |  |
| - 1. Describe the physical location of the HIV, diabetes and hypertension clinics.      1. Where are each of the clinics located (refer to photo or floor plan);      2. Where is triage of patients performed? |  |
| - 1. Describe the waiting area      1. For HIV clinic      2. For NCD clinic |  |
| 1. **Description of the service provision** | |
| - 1. When are services for HIV, diabetes and hypertension provided? Include day and time. |  |
| - 1. Are any services co-located/ delivered at the same service point? Describe which services are provided together.      1. If services are co-located, is there a common triage point for both clinics? |  |
| - 1. Drug dispensing      1. Does the health facility have a pharmacy (observe the privacy)?      2. Where do patients wait while collecting medication? |  |

**SECTION TWO**

| 1. **Observations about the waiting area (for registration and clinician consultation)** | |
| --- | --- |
| - 1. How is this organised? |  |
| - 1. How comfortable are the patients when entering the clinic (check active, shy, unhappy or normal through facial expressions) |  |
| - 1. How comfortable are the patients with the seating arrangement (check active, shy, unhappy or normal through facial expressions)? |  |
| - 1. How free are they to discuss anything with their neighbours while at waiting area? |  |
| - 1. What is the rapport between patients and care providers (from reception, facial expressions, language among and between them etc) |  |
| - 1. How comfortable are the patients when moving from one section to another (consultation room, laboratory, pharmacy etc.)? |  |

| - 1. How comfortable are they in picking up the drugs in the eyes of different people (the package/boxes of some medicine eg for HIV are easily identified and might necessitate unpacking for easy carrying) |  |
| --- | --- |
| - 1. How are the instructions on medicine usage are provided (secretly with low voice/tone, louder, normal) and how do the patients react to the instructions (shy, happy, normal)? |  |
| - 1. Is there any health education or counselling provided as patients wait (observe the response of patients in terms of listening attentively, asking questions etc)? |  |
| - 1. Are there any other activities that patients have to attend while at the clinic e.g. patient meetings, exercise groups etc.? |  |
| - 1. Are there any expert patients involved in patient education or patient meetings? If so, describe what they do and how other patients respond to this. |  |
| 1. **Any other observations** | |
