# Supplementary material for: Integrated healthcare services for HIV, diabetes mellitus and hypertension in selected health facilities in Kampala and Wakiso districts, Uganda: A qualitative methods study
Source: PLOS Glob Public Health. 2022 Feb 3;2(2):e0000084. doi: 10.1371/journal.pgph.0000084 (PMC10021152; doi:10.1371/journal.pgph.0000084)
Supplement: S1 Data — (DOCX) [file pgph.0000084.s002.docx]

MOCCA Data Analysis framework; How did the integrated care delivery model affect access to HIV, DM and HT care services? (service users)

| Variable | Phase 1 | Phase 2 | Phase 3 |
| --- | --- | --- | --- |
| **Availability** | **OVERVIEW**  **Strengths:**   - HT, DM and HIV screening services (Measurements; Blood pressure. Height, weight / blood screening in the Laboratory) - HT, DM and HIV Drugs availability - Well treated by the health workers - Health workers’ availability /Good interpersonal relationship   **Limitations:**   - Unavailability of some drugs i.e. HT and DM - delays at the facility. - Facility being understaffed. - Use of herbal medicines - Unavailable drugs - Poor adherence due to unavailable drugs | **OVERVIEW**  **Strengths**   - Availability of Drugs - Good quality services - Procedures and provision of Testing services: - Availability of health workers and their attitude towards patients - Waiting time was sufficient - Availability of Health workers - Interpersonal personal relationship - Stigma Reduction   **Limitations**   - Unavailability of some drugs - Delays at the facility | **OVERVIEW**  **Strengths:**   - No time wastage - Availability of drugs - Good quality services - adherence - Well treated by the health workers and their availability - procedures and time spent at the facility at the facility   **Limitations:**   - Delays at the facility - No privacy - Stigma at the facility - Drugs unavailable |
| limitations | **Unavailable Drugs:**  Most of the patients especially those with HT and DM find a challenge of some drugs being unavailable at the facilities which is not the case with HIV drugs which are always available at all times. Apart from a few patients who present with missing septrin drugs some times. Most of these patients are always advised to purchase the missing drugs with money from their pockets which some they may not have.  **40-50 year female DM /HT Patient:** ‘’*sometimes drugs are unavailable at the facility. When I don’t have money, I don’t buy enough drugs, I buys just enough to take me for some time and then I run out of money and drugs and I will wait until I gets money to purchase other drugs and that has been my greatest challenge’’*  **30- 40 year female HT Patient;** *‘ ’There is a moment I came and found one type of drug unavailable, I was asked to buy it elsewhere, this means that some drugs are not always available at the facility*…’  **30-40 years Female DM Patient***: ‘’Treatment has been good, just that there was a month we used not get drugs at this clinic because the drugs where not readily available at the pharmacy’’*  **30-40 year Male DM Patient:** *‘’I don’t know why the facility can at times have stock outs and i can’t ask why because it would appear impolite’’.*  **A lot of Time spent at the facility**  Almost all patients complained about spending a lot of time at the facility, they state that they go through a lot of procedures and spend most of the time at the pharmacy in a que for drugs. They described the procedures, as  soon as you arrive you report to the triage for registration since it is first come first serve so whoever comes first will be the one to be served first. They then proceed to the triage where measurements are done then go to the integrated clinic where one sees the health worker to asses her or is condition after there you may be sent to the laboratory of given you prescription and proceed to the pharmacy there after one goes home.  **50-60 years female DM/HT patient*:*** *‘‘I* *spend most time at the pharmacy and the least at the doctor’s consultation room, the triage and the lab for bleeding’’*  **40-50 years male HIV patient**: ‘’*I usually delays much at the pharmacy when I am picking drugs because there are always many people’’.*  **30-40 years Male DM Patient:** *‘’‘What cause the delay at the reception is because there is only one care provider attending to an overwhelming number of patients alone. This is what cause the delays’’*.  **Ruthless treatment of patients by health workers:**  Some health workers have not been supportive to patients, some of them were barking at them so they faced a lot challenges blending with them.  **60-70 years male HT patient: ‘**’*One of the health workers i went to consult from angrily asked me to go and queue on another section, it is there then that i met another health worker who attended to me.*  **60-70 year Male HT Patient:** ‘’*I think the quality of service was wanting since i did not get enough attention and drugs. I even asked to be bled but i was asked to come back the following day. I was only done blood pressure measurement. I did not have good reception with the health workers, I even wanted to explain to one of the health workers i found in a room and there was no privacy at all and i wouldn’t and the health worker asked me to leave and go to another room. From there i was already demoralised, the doctor also instead of giving me drugs, just wrote down the drugs for me and i did not have money to buy them’’.*  **Lack of enough space for patients at the facility:**  **60-70 years Male HT Patient;** *‘’i thought that the facility was not good enough for treatment since it did not look like a hospital. I compared other hospitals which give similar services such as Nsambya hospital. The facility lacked many resources, the place where to sit was small and there was not enough for others, there is need to improve on the infrastructure’’.* | **Unavailable drugs;** There is always unavailable drugs especially for the HT and DM patients and at Wakiso they have a drug group where they pay some subscription fee every month and they get drugs collectively.  **40-50 years Female DM Patient*:*** ‘’*We pay some money to some group at the clinic through which I would pick my drugs and if I did not pay the money, I would not get the drugs. I pay 30,000/- to the group i.e. subscribed to at the facility, and even paid some money for the drugs, I pay money according to how much drugs I am going to go home with. In effect, I pay for the drugs I carry home and on my last visit, they gave me very little drugs because I was not able to pay the full money and once I am given little drugs, I stop taking drugs and sometimes if I don’t take the drugs, I would cut down on my dosage…’’*  ***40-50 years Male HIV Patient:*** *‘’My biggest challenge on drug availability was the lack of septrin, I only accessed septrin tablets when I had just been enrolled into care but ever since I enrolled, I have been asked to always buy the drugs which I do at the clinics of private pharmacies’’.*  **Delay at the clinic;**  **60-70 years Female HIV/DM/HTN Patient:** *Some days when I come late, I usually leave at 1pm especially when they delay to give me drugs from the pharmacy, I was not aware as to why the drugs take a lot of time before they are dispensed to the clients.* | **Stigma at the facility:**  **20-30 year female HIV patient:** ‘*’it is disturbing getting care with other patients, you always get that ill feeling, you imagine you are sharing a seat with someone who is diabetic yet you are HIV positive. You feel stigmatised with so many questions but is still comes back to you encourage yourself because there is nothing you can do about it and these conditions are chronic*’’.  **70-80 year female DM and HT patient*:*** *‘’my only concern came with the fact that sometimes I may sit among people who were infected with TB and get me sick’’*  **Delays at the facility:**  **40-50 year male DM patient ‘***’The only place where he found difficulty in the whole process was the lab as they make him wait for so long to get a service’’.*  **30-40 year female DM patient** ‘*’I spend much of my time at seeing the doctor. This process is longer because there is only one doctor yet the number of patients is very high. It is this doctor that sees all patients apart from those that are HIV positive’’*.  **Unavailability of drugs**  **30-40 year female DM patient;** ‘*’The quality hasn’t been bad, it is just that there are no drugs at times or if you are to get 2 types of drugs you will find that you have only gotten one type and the other type is not available yet you will have spent so much time waiting for drugs just to be told that the drugs are not available’’*  **No privacy:**  **30-40 year Female DM patient**  You can use the card to tell if they are going to see the same physician like you are or not. We have pink cards and they (HIV patients) have blue cards and that’s what you use to tell that he is going in the other direction and I am going in this other direction. Diabetic and hypertensive patients go in the same direction and HIV patients go in another direction. |
| Strengths | **Availability of drugs at the facilities;**  This was mostly pointed out by HIV positive patients who say that there is always availability of drugs at the facility at all times which is not the same with HT and DM patients who complain of unavailable drugs. Patients at some facilities like Wakiso HCIV have formulated their own clubs with the aim of buying drugs in bulk instead of individually this helped them in reducing costs of treatment and availability of drugs to those in the association.  **40-50 years Female HT&DM patient**: ‘’*This association was formed because the drugs are not always availed by the health centre so the patients contribute money and buy the drugs in bulk then whenever they come for drug refill, they get their medication from the association pull’’.*  **Availability of drugs**  **50-60 years Female HT Patient:** ‘’*The medication is available most of the time and when it is not available. I am told to come back another day for it. I contribute money to buying these drugs (through the association), the amount I contribute depends on the combination of drugs one takes. It can be 30000, 40000 or more.*  ***40-50 years male HIV****: ‘’when I come to the facility, there was no day i found when there were no drugs and I would pick drugs whenever I would come over and all of them’’.*  **40- 50 years male HIV:** ‘*’according to the other facilities i have been too I feel the facility is good and all things are organised’’.*  **30-40 years female DM Patient*:*** *‘’Today I got all the three types of drugs that I use every after one month and it has only happened twice when I visited and found there were no drugs but even then I could get one type and could not get the other type’’.*  **Convenient with the procedures are friendly and affordable:**  **50- 60 years female DM/HT**: ‘*’The procedures I take to pick my drugs are very convenient and friendly and i don’t have a lot of difficulties.*  *The services i receive are good especially with the health talks in which they are told to refrain from some actions, some food, drug adherence and counselling’’.*  **30-40 years MALE DM/HT:** ‘’*health workers are very clear with the information they give us and I am happy about it… I am very comfortable with the procedures which I went through to access drugs’’*  **Relationship with Health workers**  *Patients who visit the facilities have asserted that there is a good interpersonal relationship with the health workers reason that they could even call them and have them reminded about their next day’s appointment so they wouldn’t expect more concern than where the care provider calls the patient.*  **30-40 years male DM Patient:** ‘’*We were also told while being counselled that we shall all receive the same medication whether you have one condition or several of them so I am certain those other patients are also catered for’’* | **Availability of drugs**  ***60-70 years Female HIV/DM/HT Patient***: ‘’*I have been getting good treatment and care from this facility ever since they made it an NCD centre, because whenever I come to the facility, I get all medication for all my conditions and I do not go to other facilities or clinics. Ever since I started using the integration system, things have become so easy for me because I no longer have to access drugs from other sites’’*.  ***30-40 years FemaleDM Patient:*** *‘’ I have only missed once ever since I started getting drugs from here and even then I was told I would get drugs the next time I return. I use to types of drugs, nimepride and metrofomin. I have been getting these two types for all the time I have been coming to the facility. They were actually three types but I was put off one and left with two’’*.  **50-60 years female DM patient:** *‘’About the availability of the drugs, drugs for diabetes which is my condition are readily available and in plenty. I only missed once when I was starting, there were some tiny drugs that were not ready available and I had to buy them, they costed me about 30.000 but they have been made available now’’.*  **Waiting Time is sufficient:**  **30-40 years FemaleDM Patient*:*** ‘’*The waiting time depends on the number of people you find here, the more the people is the longer you will spend here and vise-viser. Otherwise some time you can even live here in about 30 minutes so the time is reasonable she said when asked about the waiting time’’.*  **40-50 years female DM patient:** *But for the integrated clinic, I do not take a lot of time waiting to be served, I could spend two hours waiting to be served which is very convenient and for all the procedures and where to go to pick a service, I was very much aware exactly what to do and where to go.*  ***20-30 years Female HIV patient*:** ’The waiting time is just enough reason being that by the time you leave you feel relieved and contented. For as long as someone has come in early enough, they will always leave in time as well’’.  **60-70 years Female HT&DM patient:**  *According to me, the sessions were not delayed. As compared to the former time, I no longer wait a lot. I suggest that the best waiting time would be waiting at least four hours that was two hours to wait for the health workers and two hours while receiving medication.*  **Good Quality of services:**  **40-50 years female DM Patient:** ‘*’On the quality of services, the way DM was handled was fairly good although I did not get other services especially for the other diseases that I come with’’.*  ***20-30 years Female HIV patient****: ‘’I started swallowing drugs in December 2018 but I think I joined the integrated clinic in February 2019.There is good treatment and care in this facility, even though it so happens that sometimes I miss my appointments but I feel it is better and relieving to talk and share with a care provider as opposed to suffering in silence but even when you come in at any point in time even if out of your appointment, they will still receive* you very well’’  **60-70 years Female HT&DM patient*: ‘’*** *I was given quality service, it was orderly, there were no many people and the whole session according to me was not delayed. I had a good relationship with my fellow clients and we discussed a range of things’’.*  **Availability of Health workers**  **50-60 years HT&DM patient*; ‘’****The health workers are very responsive to the needs of the clients and are always supportive whenever there is need’.*  ***20-30 years Female HIV patient*:** ‘’Everything was on point today. I personally came thinking they were going to back at me because I had missed my appointment but because I started by apologizing to them on reaching the facility’’  **48yrsfemaleDM patient:** ‘’*I always find health workers at the facility and the laboratory was good for her the treatment of my condition. I am very comfortable with the health workers and the way they treated us. The health talk we had which discussions a range of things such as drugs adherence and treatment and other diseases’’.*  **Interpersonal Relationship:**  **50-60 years female DM patient*:*** *‘’The interpersonal relationship is also fine because they want to know how I have been taking my medicine, how I am fairing, any other complaints that I may be feeling so the relationship is truly good’’.*  ***20-30 years Female HIV patient****: ‘’I started swallowing drugs in December 2018 but I think I joined the integrated clinic in February 2019.There is good treatment and care in this facility, even though it so happens that sometimes I miss my appointments but I feel it is better and relieving to talk and share with a care provider as opposed to suffering in silence but even when you come in at any point in time even if out of your appointment, they will still receive* you very well’’  **Stigma Reduction:**  ***20-30 years female HIV Patient:*** ‘’some people wouldn’t have wanted to sit next to other people having in mind that those particular patient’s condition is infectious but little did they know that it wouldn’t infect by body contact but they were later explained to and they opened up’’.  **60-70 years Female HT&DM patient**: ‘’*I have never got any challenge accessing drugs and going through the whole process of accessing drugs. I cannot even get any form of shyness or be belittled when I am going to pick my drugs because I personally did not even know where exactly the persons with HIV where seated and this means there was no stigma of any form or kind’’.*  ***20-30 years Female HIV patient*:** ‘*’There are at times I would feel so inconvenienced especially at the start. I could literally quake on being asked to go to a particular service point often times I would be tempted to think these people were not being mindful of me but I have now settled in and I always encourage myself because the health workers won’t always be there to do things for me that I am meant to do for myself. I used to fear so much and I would frankly tell the care providers that I wouldn’t be in position and that I needed then to help me, I usually asked Isaac (the health worker) to help me pick or reach certain points because I was afraid of being seen’’*  **40-50 years female HIV patient**:  ***20-30 years Female HIV ‘’****Actually people used to fear coming to TASO because they used to think it was a one stop centre for HIV patients but this stigmatization is slowly fading away plus, the drama skits that are performed here by us the expert clients make some patients to come along with other fellow patients to come and test and also get treatment’’*.  **Health education**  **60-70 years Female HT&DM patient** *They usually get a health worker from the district who also comes and conducts health education talks. According to her, she told me that she has been able to benefit a lot from the health education which she gets because, it has enabled her to reduce her sugar levels from the information she gets.*  **Procedures and provision of Testing services:**  **40-50 years femaleDM patient:** ‘’*I wait at the waiting area and the health workers give us a health talk, from there with others, tests such as blood sugar level tests, blood pressure and weight plus height are all done on me. Blood is drawn for DM and since she has DM, at the laboratory and from there, then we to the health workers for diagnosis and examination.*  ***20-30 years Female HIV patient*:** I am always here at about 08:00 o’clock because usually we first have some health education sessions first before the health workers begin examining and further working on us. Our weights are taken and then we get in a one on one session with the care provider where after which we go pick our medicine and leave  **Adherence:**  **40-50 years female DM patient**: *She said that she had been getting drugs and not missing them ever since she joined the integration. The whole process of treatment according to her was very convenient and easy for her to go along with since, with the general clinic, the process required a lot of patience, waiting with so many clients, hunger bites and yet even the health workers don’t come early as compared to the integrated clinic*  **20-30 years Female HIV patient ‘’**I have been so committed to adhering because they have taught us so much that makes us to taking our drugs. Sometimes you lose hope but they give you advice and counsel and you will feel contented about your new status and start taking drugs’’. | **Procedures and time spent at the facility**  The procedures don’t take long since they know where and what do to do than it was before. They appreciate the procedures and the time they take at the facility  **50-60 year Female HT Patient:** *‘’I am always through with all the procedures at the facility by mid-day although that depends on how one comes early. It was so easy for me to receive medication as I always come earlier and I get served’’*  ***20-30 year Female HIV patient:*** *it saved us a lot of time wastage, lately we no longer have to keep moving from one point to another, we almost get all services in one place in the integrated clinic so it is less inconveniencing. Once you are done seeing the health worker at the integrated clinic, then you are either headed for home or for your drugs refills at the ART clinic*  **40-50 year Male DM patient: ‘*’When*** *he comes to the facility, he starts at the integration table and triage, from there, he goes to the health worker, and the MOCCA team has always been making it easy for him to move swiftly. He told me that since he had DM, it was always easy for him to go through this whole process efficiently. The only place where he found difficulty in the whole process was the lab as they make him wait for so long to get a service’’.*  **Good quality service:**  **50-60 year Male DM/ HT patient:** *‘’I find service delivery good except that we are not given something to eat but we get all the medical treatment’’.*  **20-30 year female HIV patient: ‘’** *the integrated clinic has helped us in a such a way that you get to talk with the health worker about certain things in life for example those that have any condition will attest that there are times you wake up with something bothering you and you are left with consulting with the health worker what might have possibly caused it. They will always have you counselled if you were not leaving your life right and they will ask you to stop doing certain things if you have been doing them. They explain to you and you leave the facility contented and relaxed’’.*  **20-30 year female HIV patient:** ‘’ *They treat us so well in that even if you do not have a plan of coming back to the facility you will force yourself to come to the facility because of the way they handle us and the care they give us’’*  **Health workers’ availability;**  **70-80 year female DM/ HT Patient:** ‘’the health workers spoke Luganda to me and were always very supportive and always give them health information which helps me to stay away from other fatal conditions’’  **No stigma:**  **40- 50 year male DM patient:** ‘*’there was no more need to fear when one had a NCD condition; this was because all were created by God and if one was sick, they had to find medicine. He told me that he had felt a very big change in his treatment regime. And all the service points were all good, but especially at the triage and at the pharmacy. They would work on him faster and well at those points’’.*  **Adherence:**  **40-50 year male DM patient:** *On adherence, I have been very faithful to my regimen since it was the only way I would be well and be guaranteed of a good life. I have never got any side effect or challenge related to his drug, but for missing, I have once missed one my appointment because of the nature of my job.*  **Procedures**  **50- 60 year male DM/HT patient**; *‘’I get at the facility, I go straight into the integrated clinic from where they take me for an RBS then they bring back the results and on noticing that the blood sugar is unstable, they will send you to the laboratory and still bring back the results to office from where you get to learn in more from the health worker about your condition. I think I have spent about 2 years while getting care under the integrated clinic. I don’t think I have been under the integrated clinic for more than 2 years’’.*  **Procedures**  **50-60 year male DM/HT patient**; *‘’I don’t feel inconvenienced following these procedures, at least not anymore, I do not feel ashamed or even fear the people who come from the same place as I do- I am so used to that anyway’’.*  **30-40 year DM female patient:** ‘*’I personally have no problem with it for as long as I have gotten my drugs or for as long as I see my doctor for review then I get to know my current diabetes reading and I have gotten my drugs, the rest is not my business whoever you make me sit next to for as long as I am not seated next to someone who has TB’’.*  **Availability of drugs / treatment**  **50-60 year male DM/HT patient**; ‘*’It was good because I used to come to the facility and they could still prescribe the drugs for me and ask that I go and buy it from elsewhere. I get free care and treatment as well now unlike back then. There is a good relationship between me as a patient and the care providers plus, the medical team makes sure that they speak to you in a language that you best understand, any medical person will show you the love that you need in regard to the type of work s/he is doing not outside so that what happens here’’.* |
| **Affordability** | **Strengths**   - Availability of services like screening and treatment free of charge - Waiting time was okay for her - Less time for waiting at than facility for treatment than before - Services are provided free of charge so they are affordable to all community people.   **Limitations**  Transport challenges | **Strengths**   - Transport affordable   **Limitations**   - Transport costs - Unavailability of food | **Strengths:**   - Transport affordable   **Limitations:**   - Transport costs |
| limitations | **Transport a challenge:**  Some patients have missed drug refill due to lack of transport to the facility since some of them come from very far away from the facilities where they pick drugs and in case they find that some drugs are missing yet prescribed they then have to buy these drugs. This in return leads to poor adherence to drugs and thus heath deterioration.  **30 – 40 years Male DM/HT Patient: ‘’** I sometimes fail to get transport to the facility for treatment*. I came with a boda boda which cost me 2000 to come to the facility and i was thinking of walking back home in the guise of doing exercise because there was no money I had at hand’’.*  ***50-60 years Female;*** *‘’Since I started receiving treatment for hypertension, I have never gone off drugs however sometimes I miss my appointment days because of lack of transport to the health centre’’.*  **40-50 years Female HT&DM patient***: ‘’ I get my drug refills for both conditions on a Thursday with those with diabetes but because I have a combination, I come to the clinic on a Wednesday to learn some things from those with hypertension because they might learn somethings about hypertension that I don’t get to learn with those of diabetes’’.* | **Transport costs;**  ***20-30 years Female HIV patient*:** ‘*’It would cost me about 40.000 from my work place to Uganda and about 5.000 more to get me to the facility. So it costs me between 45.000 to 50.000 shilling to come with no return journey’’.*  **40-50 years Female DM Patient*:*** ‘’*The journey I make from home to the clinic was very long and I also cited that I had challenges at home with my husband. I said that I did not have money to pay for my transport to come to the facility and yet my husband who provides for me makes a strict budget for my expenditure’’*  **40-50 years Female DM Patient**: *I spend 4000/- to come to the facility and yet there was no clinic in my new home area. I however have a small clinic in which I could go and have some blood tests although I did not do it occasionally as it would take some time.*  **Food unavailable:**  **50-60 years female HT&DM patient:** ‘’*Among the challenges that I get while taking my drugs is the absence of food, my drugs require a lot of food, and once I take drugs without food, I become dizzy, shiver, becomes weak and sometimes passes out.* | **Transport costs**  **20-30 year female HIV patient: ‘***’ I use 7000 to 10000 Ugshs every time I have to come to the facility. I have ever missed appointment because of not having transport but it made me feel bad missing my appointment on grounds that I lacked transport and I could not foot all the way to the facility but I promised to go and explain to the health worker’’.*  **50 -60 year female HT patient**: *‘’ I have difficulty with the money for transport as I don’t make as much as I used to make many years ago having been chased from the city centre by the city authorities’’* |
| Strengths | **30-40 years femaleHT Patient; ‘’***I expressed my gratitude towards the integration because formerly my HTN drugs were very expensive I would fail to purchase them’’.*  **40-50 years male HIV Patient:** ‘’*… the waiting time was not that bad on the day for our chat and I also hinted out on the rain as having facilitated the low waiting time for the day. I use around 30 minutes to walk to the health facility and it was not far, I even thought that it was good because it would make me do some exercises. Although sometimes I use a bodaboda which costs around 1000 shillings of his money’’.*    **Appropriate Waiting time**  ***30-40years Male DM Patient:*** *‘’Time is fair enough and has been fully utilized if you compared it with how it used to be back then. I used to come here at 8 in the morning and I could leave at 3 in the evening’’.* | **Transport affordability**  **40-50 years male HIV patient:** *‘’When I use the bodaboda, I don’t get an extra charge, and I only use it if I wake up late. I use 2000 shillings for the whole joinery and there is no extra money I pay to access any medication or treatment’’.*    **60-70 years HT/DM/HIV patient:** *Since I pick drugs after a period of two months, I don’t have any constraint with getting transport money. Medication is free at the facility and there has not been a day when I came to the clinic and did not find drugs available.* | **Transport affordable**  **20-30 year Female HIV Patient**: *’’I find the distance factor as no issue unless if I am being asked to come here on a daily basis but if not then I have to always keep it at the back of my mind that always have to make a day to come to the facility for the sake of my life’’* |
| **Acceptability** | - No difficulty in following procedures - comfortable with the integration - waiting time okay for her - information used to be unclear during the days of no integration - Services at the facility are commendable | **Strengths:**   - Integration acceptability - No Stigma - Continuity   **Limitations:**   - Long waiting hours | **Strengths:**   - Good interpersonal relationship - No stigma - Ready to use the integration clinic - No stigma   **Limitations:**   - Feeling stigmatised - Fewer Health workers / long waiting hours |
| limitations | **Stigma**  **40-50 years male HIV**: ‘’*those afraid and have stigma, it may not be easy for them and some may even confuse others for having HIV because this is an AIDS facility’’*.  Some patients pointed out their concern for the integration as they expressed fear of giving patients wrong medication since they will be in same clinic with different illnesses with the integration in place.  **50-60 years female HT patient: ‘’***I fear we may be given the wrong medication like being given medication for HIV patients*. *Because we will be in same clinic with HIV patients’’.* | **Long waiting hours at the facility:**  **40-50 years male HIV Patient:** ‘’*For the health workers, they don’t come early and especially at the pharmacy, they could work on all of us, when they all came early but the pharmacy would still be closed and we have to wait for long hours to pick their drugs’’.* | **Feeling stigmatised:**  **20-30 year female HIV Patient:** ‘’*following the procedures makes you feel stigmatised at times more so if you just started with the ART treatment but you get used as time goes by. You get to learn that you are saving your life, there you stop being mindful of other people’s opinions because all you are doing at that time is to try and save your life’’.*  **Fewer Health workers and long waiting hours**  **30-40 year female DM patient:** ‘*’The only challenge it comes with is seeing the doctor. We have one doctor who works on all of the patients in at the facility. It is this doctor that works on those that are vomiting, those that are young, the elderly, those that are hypertensive and those that are diabetic. So if you go without money to buy something to eat might make you uncomfortable because you might spend longer while hungry but the rest of the points of kind of changed albeit’’.* |
| Strengths | - It is very easy for him to follow the procedures at the clinic because the health workers are very clear with the information, the rules as he called them which are meant to help them pick the drugs. - Movement made easy for those with multiple conditions. - Integration will be very helpful to improve the quality of services   **Reduced stigma due to integration**  **30-40 years female HT Patient; - ‘’***clients will be able to become more in the integration scheme because she thought that there was a lot of publicity and response from the communities. stigma among persons with HIV was coming down and people would not be afraid to go and pick drugs from a centre giving medications to three conditions’’.*  **Relationship with health providers**  **30-40 years male DM/HT Patient:** ‘’*I applaud the relationship I have with the health workers who are very friendly, unlike those in Naguru where the relationship between the patients and clients is very rough.* I am aware that the cause *for integration was that, all the conditions that are similar are made to have one docket with which they can be worked under one health centre. He was willing to continue taking drugs under this arrangement. He feels the integration is good since they are made to do things in procedures and very fast.*  *unless one gets a disease different from others’’.*  **Adherence**  ***40-50 years male HIV Patient:*** *‘’He had never missed any of his appointments and when he is home, he makes an effort to take his drugs on time. He has a form of a calendar with which he uses to tick each time he takes the drugs’’*  **30-40 years Mal*e DM Patient:*** *‘’ I have been adhering to taking my drugs as well as getting my treatment and following all the procedures given to me by the health team for example, I make sure I honour all appointments that I will have been assigned. There won’t be any reason to deter me from coming to get my treatment even in as much as my kind of work doesn’t allow for me to get enough time to exercise’’*  **40-50 years male HIV Patient:** ‘’*He has been able to disclose to his son and his sister about his HIV status with whom they stay in one compound*. *He told me that they support him by reminding him about when to have drugs on his appointment, they also ask him not to involve in heavy duty jobs that require a lot of physical input’’.*  **50-60 year Female***:’’ I always come to the clinic and do exercises and learn about the foods to eat. However, I have finished like three months without coming for the health talks but I exercise from home. The health workers also talk to us on clinic days about exercising, taking drugs in time and things like that’’*  **40-50 years female DM/HT Patient***:*  **‘’***She also told me that, she never found it difficult to follow the procedure because her intention was to have drugs, she did not mind any way that she would be asked to follow the procedures’’.*  *‘’according to her, she would be shy if one had a different disease as compared to others. But when she discovers that one has HT or DM and all of you have one similar disease, one may not get shy’’,*  ***Quality of Care***  Patients finds the quality of services at the clinic good. This is because they are now different from the time they joined the clinic.  ***30-40 years Male DM patient****: health workers are well elaborate and provide them with opportunity to ask questions in case something was not clear for them.*  *Some of the advantages I have enjoyed being under the integration is quality care and good interpersonal relationship between the patients and the care providers that is given at this clinic*  **50-60 years Female HT patient** *‘’ I joined the clinic when I was badly off, I was sickly but now I feel fine. my pressure is also normalising in that even the medication I was using then was a lot and more expensive but they changed my medication too*.  **30-40 year female HT Patient; ‘’***The clinic gives very good standards to the health workers, this is because when she came to this hospital she got the same better services that she was receiving elsewhere with all medication available’’*  **50-60 years female**:’’ the standard of services is good and it is reasonable according to my estimation and desire *I have been able to get tests for my kidney problems’’.*  **50-60 years female HT patient**: ‘’*maybe the integration will improve the quality of services at the facility because I don’t think after getting what we want from them, we will leave them without doing anything for them. For now, she hasn’t seen any drugs or anything but just waiting to see what we shall do for them’’.*  Patients finds the quality of services at the clinic good. This is because she is now different from the time she joined the clinic*.*  **50-60 years Female***: ‘’ I joined the clinic when I was badly off, I was sickly but now I feel fine. my pressure is also normalising in that even the medication I was using then was a lot and more expensive but they changed my medication too*.  **40-50 years female HT&DM Patient:** ….*the quality of services received at Wakiso HC IV is good because sometimes patients come from Kirundu to come and receive treatment at Wakiso HC IV saying they have been told there is good treatment for hypertension and diabetes at Wakiso HC IV.*    **Willing to join the integration**  **50-60years female HT patient:** Many patients have shown interest in the trail which means they will be willing to get treatment in an integrated clinic. I initially wanted to join the trial because I thought MRC may be giving out free drugs but later I decided to join hoping I might benefit in a way in the future. | Integration acceptability;  **60-70 years Female HT&DM patient:**  *she was very comfortable with the integration since it also gave her time to come at her own time and that since she does not eat when she is coming to the facility, she finds time to have something to eat once they have finished giving her medication in the morning*  **Integration acceptability:**  **40-50 years male HIV patient:** ‘’*I came to know about the integration when I came to the HIV clinic, I was asked by one health worker who introduced the integration scheme to me, he was however not so conversant with the scheme although he managed to tell me that there were three conditions that where treated which included HT, HIV and DM. he told me that the integration was good because if one came with so many conditions, they would have their treatment and drugs in just one day. Among the things I have gained from the integration was, receiving drugs for all the conditions, quick service and sometimes availability of drugs and no payment to receive any service’’.*  **60-70 years HIV/DM/HTN Patient:** ‘’*According to my observation I knew that people would love the integration since, it started with small number of people but other sections of people have started embracing the integration and there is a steady number at the facility, the integration would help reduce stigma since these days people can only know of their status once they are tested and it was only long time ago when people would hide because they did not know their respective status. There was no more hiding anymore and among my fellow clients where those who came from my home area and yet they were receiving treatment from one similar health facility*.’’  **30-40 years Female DM patient:** ‘’*I like the integration idea because it has helped me as an individual and other patient to live because it would otherwise have been very costly yet there are times when I sincerely do not have the money.*  *I am willing to continue getting care and treatment in such an integrated model, because I have to live and my life depends on it so I am more than ready to continue under the integration’’.*  **50-60 years female DM patient:** *‘’I think the initiative is good because it has helped me otherwise I don’t know where I will have gotten the money to buy all this medicine in this day and era. Here we are prioritized, I don’t know why but the arrangement is favourable and affordable for people with diabetes and hypertension’’*  **40-50 years female DM patient**: ‘’*I have been getting drugs and not missing them ever since I joined the integration. The whole process of treatment according to me was very convenient and easy for me to go along with since, with the general clinic, the process required a lot of patience, waiting with so many clients, hunger bites and yet even the health workers don’t come early as compared to the integrated clinic’’.*  **60- 70 year Male DM/HIV/HT Patient:** ‘’*One of the good things I have got from the integration is that, once someone is not doing fine, she could free information on what to do, I would get to know about the nutrition I am supposed to use for my better health and the time and money I used to spend moving from one place to another to pick drugs has also reduced. I would formerly pick her drugs from Mulago very early and I would go back without drugs and also when I am very tired. The integration has also reduced the amount of money and time that I use at the facility since I no longer spend any more money going to other facilities’’.*  **Good relationship among patients**  **40-50 year female DM patient**: *there was a good relationship among the clients and we supported each other very well. I had not seen anything bad about the integration as far, but only that there should be enough space for the clinic.*  **NO STIGMA**  ***60-70 year Female HIV/DM/HTN Patient****: I did not feel any form of stigma when I came to the facility because all of those who did come to the hospital were patients, so even though one pinpointed to me about her illness, it would not make any sense since nowadays, you may not know who is sick or ok unless they tested for any disease.*  ***Continuity:***  **40-50 year female HIV patient**: ‘’I would wish for the idea to spread to other facilities because it is only in a few uptown facilities. People in the villages should as well get to enjoy such services. I am willing to get my treatment through an integrated clinic and I am not ashamed of getting care under such an arrangement’’.  **Comfortable with the service points:**  **50-60 year female HT&DM patient:** ‘’*I am very much comfortable with the way I am served and commended all the service points as being friendly and supportive*…… *the integration was a very good scheme and I was in support of it although I did not know why it was good’’* | **Good interpersonal relationship:**  ***20-30 year Female HIV patient:*** ‘’ *there is good interpersonal relationship between us and the care providers they always explain to us and you will indeed feel contented by the time you leave the facility. They explain in the language I understand and with a lot of care and concern’’*  **20-30 year female HIV patient:** ‘*’It was very obvious back then for someone to tell that you have such a condition because we used to get care from different stand-alone clinic at which whoever goes to is labelled depending on the service offered at that clinic. After the integration, you know the idea might not be working for some people in a sense that if you had a particular condition, then you had to seat and get services in a particular clinic and you could not draw to close to one another but lately we sit together and even chat some times’’*  **20-30 year female HIV patient:** ‘*’ I am willing to getting care in the integrated clinic. as I told you they will always have reservations but there is nothing you can do about it. Such reservations might even be with in you. You will at times feel weakened at some times when you get at the facility but you stand strong and get my treatment’’*  ***Benefits of integration;***  *20-30 year female HIV patient: ‘’ I used to miss appointments at the very start because I would convince myself that there wasn’t a lot I was missing after all I had some balance but I am always encouraged to come lately because of the care I get and the way they have me counselled’’.*  **30-40 year female HT Patient**: *‘’The MOCCA team was very caring, they spoke to me in a language which I understood, since I did not know Luganda, they cared to speak to me in both Luganda and English. ..……. I was comfortable taking drugs from a clinic which treats other conditions. I benefited from the integrated clinic as I got treatment, there was commendable care, and drugs. The integrated clinic would help me to also check some other ailments such as HIV and DM’’.*  **STIGMA**  **70-80 year female DM and HT Patient: ‘***’ I have never felt any stigma and this was because I sat at the MOCCA study room where not many people converge. I did not even feel any shyness walking from the MOCCA study room to the pharmacy or lab to have services there because i knew that I did not have HIV and would not mind if any one said i had’’*  **Appreciation of integration:**  ***50-60 year female HT patient:*** **‘’I** *am very positive with the integration it has helped me live a worthwhile life I received drugs on time and I get information which wold have been difficult to get free of charge’’*  ***50-60 year female HT patient:*** *‘’the integration has been very power full symbol in my life formerly I had HT but was not very active taking my drugs, but once I joined the integration it gave me a =boast in my health. I invited my friends to come to the integration I was only sad because I found when the integration stopped recruiting clients’’*  ***50-60 year female HT patient: ‘****’on choosing between a standalone chronic and an integrated clinic, she told me she would choose the integration, this was because she felt these conditions move along, one could have HT then the following day, they have DM. therefore an integrated clinic would be helpful, as it would even help one reduce the amount they would spend on transport.*    **40-50 year male DM patient:** ‘*’once the integration came, my life changed for the better, I was always consistently ill but now I would go a record many days without falling ill. I did not think that the integration would bring any challenges, this was because it was fostering love and there was no need for stigma and shyness. I was however misinformed on HIV because I stated that I had been told that the NCD conditions would not cure, but HIV would’’.*  ***Choice between stand alone and integration;***  **40-50 year male DM patient:** ‘*’The integration was much better, since even one has a business, I would just have one day off to come and have all those issues sorted so the rest of the days would be dedicated to my business and not seeking treatment. I had even been asking other people to come and join the integration except for the fact that, on the day I came with someone, they told me that they had stopped registering new clients’’.*  ***50-60 year male DM patient***  ‘*’so I clearly do not understand how it is with other patients but I have absolutely no problem with it since it has served me better with my condition- health is wealth’’.*  ***50 – 60 year male DM patient***  willing to continue seeking care under an integrated model. He isn’t ashamed and or fears being seen by his neighbours  ***50- 60 year male DM patient***  I do not like to go back to the normal routine, I want to continue with the integrated clinic because here we are not given care alongside general OPD patients. There are relatively big numbers of patients turning up at the general OPD section as opposed to here at the integrated clinic.  **30-40 year female DM patient:**  ‘*’I was alone because under the general OPD treatment, I used not to know that there are so many people having the diabetic condition which actually used to make me feel bad but the integrated clinic opened up my eyes when I saw many files with all people having the same condition as I used to say but why is it that I am the only one that has diabetes yet I am still of a younger age but I later I learnt that I wasn’t the only one that was having this condition from the integrated clinic’’.* |
